# Abstracts of papers presented at the ISLAR (International Symposium on Laboratory Automation and Robotics) 1995

**DOI:** 10.1155/S1463924696000053

**Published:** 1996


Journal of Automatic Chemistry, Vol. 18, No. 2 (March–April 1996), pp. 29–57

### Abstracts of papers presented at the ISLAR (International Symposium on Laboratory Automation and Robotics) 1995

### Thirteen's a charm for ISLAR '95

**13th Annual Symposium Features New Sessions & Record Breaking Attendance.** It was a memorable year for the International Symposium on Laboratory Automation & Robotics (ISLAR '95). This year's symposium—the lucky number 13—enhanced ISLAR's reputation as the pre-eminent conference on laboratory automation and robotics, with features including: a 25% increase to 500 attendees, 95 podium and poster presentations, plus a first-ever special session focused on combinatorial chemistry.

**New Emphasis on Management & Drug Discovery Issues.** From keynote speeches and feature presentations to interactive discussion groups and technical poster presentations, ISLAR '95 provided a forum for the latest developments in the burgeoning field of laboratory automation and robotics. In addition, new emphasis on two of the most pressing issues facing scientists and laboratory technicians today—Managing Laboratory Automation and Automating Drug Discovery—was instituted. Three comprehensive sessions were conducted to accommodate presentations on managerial issues, as well as four sessions and a special roundtable discussion on the topic of automated drug discovery.

**New Application & Company Luncheons Highly Successful.** The three day symposium attracted a world renowned group of scientists that included representatives from 17 countries and 150 different organizations. To facilitate interaction between attendees, ISLAR offered two special luncheon programmes—one that grouped participants by application area and a special company-specific luncheon. Both provided colleagues, who share common interests and experience, with a forum to interact and network with peers.

As always, another symposium highlight was a reception at Zymark Center, Hopkinton, MA, that featured an introduction to Zymark's expanded customer service capabilities, including demonstrations of a customer service web-site, video-conferencing support services, plus enhanced validation and field application capabilities. The reception also featured demonstrations of the latest developments in robotic technology—Zymark unveiled a number of exciting new products, including the MultiDose Automated Dissolution Testing Workstation, an organic Synthesis System and the RapidTrace SPE Workstation.

The 1996 ISLAR will be held in Boston, MA from 20–23 October, 1996. Paper and Poster submissions are being accepted now. For more information on the symposium, contact Chris O'Neil at 508 435 9500, send an E-mail to islar@zymark.com or visit the ISLAR pages at http://www.zymark.com

**Dr James N. Little**

### PLENARY SESSION
### Re-engineering the laboratory

**Francis H. Zenie**
*Zymark Corporation, Hopkinton, MA, USA*

Our organizations are changing more rapidly than ever before—and the consequences range from prosperity—to fragile survival—to economic failure. Re-engineering is a process through which we can initiate and direct change rather than react to changes created by others. Re-engineering business is not new, yet it is timely to meet today's challenges.

The two primary forces driving these changes include:

(1) *Informed customers*—informed customers demand more value and stimulate competition. As both individual consumers and business purchasers, we are better educated and more demanding about receiving value for our investments. As providers of products and services, therefore, we must re-earn customers' business by providing innovative new products and greater value.

(2) *Information Technology*—IT is both a primary cause and an enabling technology helping to adapt to change. IT enables organizations to shift from inefficient hierarchies to highly efficient teams working on high leverage opportunities. Ever increasing globalization is a strategic opportunity made possible through advances in IT.

Both of these driving forces are irreversible and relentless, that is they will never return to the past. Regulation, on the other hand, is often a political, and even emotional, driving force. We have seen many examples where the desired benefits of regulation are better attained through customer education and economic incentives.

Science cannot separate itself from business and economics. Science creates the technology which drives our most robust businesses. As competitive, consumer and regulatory pressures force business to improve quality and productivity, science must lead, or at least enable, the bold steps ahead.

Our goal is to increase productivity—that is create more economic value for each unit of cost. Only when survival is at stake, must we place cost or people reduction ahead of increasing productivity. Wherever possible, people and automation should be teamed for productivity.

Mangement's role continues to change. In the early phase of the industrial revolution, managers existed to control workers who performed physical tasks. Over time, physical work was transferred to machines and workers gained more education to become knowledge workers. Expert knowledge workers became managers to manage specialized functions. Organizations were structured as hierarchies to maintain control and facilitate the flow of information. Managers focused on specialized tasks rather than whole processes which produced value. It is no wonder that serving customers got lost.
Today's managers lead and facilitate knowledge workers. Effective managers delegate work to individuals and teams and then stimulate innovation and change.

### Keynote: The new face of drug discovery with robotics and screening

**Prabhavathi B. Fernandes**
*Bristol-Myers Squibb Pharmaceutical Research Institute, Princeton, NJ, USA*

The cloning and identification of new genes as targets for drug discovery have been enhanced greatly through new methods in biotechnology and DNA sequencing. Coupled with this novel target discovery is the urgent need for the pharmaceutical industry to develop innovative drugs for new therapeutic targets. New approaches to screen design have resulted in sensitive and rapid screens which are suited to automation and robotics. Rapid identification of new leads within weeks of screen design is possible through the screening of synthetic collections, combinatorial chemistry libraries, as well as diverse natural product samples. The direct delivery of data from automated instrumentation to computer data bases has made the identification and sorting of leads possible. Through the use of robotics and automation, high throughput screening can accommodate thousands of samples per day and the identification of leads results within weeks of screening. Thus, new targets can now be cycled through screens, resulting in rapid lead identification for exploratory and drug discovery research.

### Keynote: Automation breakthrough for bioanalytical extractions

**R. John Stubbs**
*Bioanalytical Methods, Drug Metabolism, The R. W. Johnson Pharmaceutical Research Institute, Spring House, PA and Raritan, NJ, USA*

The explosive growth of analytical technology in recent years has provided the bioanalytical chemist with the power to measure drugs and their metabolites in biological fluids at extremely low concentrations. With the advent of high specificity assays, there is often little or no endogenous interference from the biological matrix. However, the biological matrix cannot be analysed directly, and the speed of sample preparation has become the rate-limiting step in achieving high throughput assays. The need for automation has now shifted away from analytical instrumentation towards the sample preparation step. In particular, solid-phase extraction has seen a major increase as the method of choice for sample preparation. It has been of particular importance in the analysis of peptides and biotechnology molecules where solvent extraction techniques that are widely used for small molecule drug analysis are not always possible.

Commercial automated sample preparation instrumentation for the bioanalytical chemist is not widely available, and the need to design an instrument specifically for this purpose became apparent. Through a series of meetings aimed at defining the process of sample preparation, and exploring all of the desirable features for analysis of biological matrices, a prototype instrument was constructed. This prototype was set up in a drug metabolism laboratory and thoroughly evaluated. Through collaborative teamwork, the instrument and its controlled software were modified and 'Rapid Trace' was born.

The initial response to Rapid Trace by the laboratory personnel has been very positive, and it has demonstrated the potential to increase the productivity of the laboratory for both methods development and sample analysis. The system has been validated as part of the analytical method, and shows superior reproducibility over manual SPE techniques for the assays evaluated.

### DRUG DISCOVERY

### High throughput screening in a small biotech environment

**Michelle A. J. Palmer**
*Genetics Institute, Cambridge, MA, USA*

High throughput screening in a small biotech group or company shares many of the hurdles found in a large pharmaceutical screening setting, but there are a number of issues that are unique. These areas were highlighted in this presentation and a perspective was given on what is involved in implementing a HTS effort.

### An automated solution for compound weighing, dissolution and distribution

**John Babiak and Linda Heydt**
*Robotics and Automation Research, Wyeth-Ayerst Research, Princeton, NJ 08543, USA*

An integral part of any pharmaceutical automated screening effort is the availability of a large number of test compounds in an assay-ready format. The most typical arrangement is to dissolve compounds in a common solvent, such as DMSO, and distribute them into microtitre plates for future use. The data generated by this process include the identity of each compound, the quantity of each compound dispensed, its final concentration in solution, the ultimate plate and well location of each compound and the volume of solution distributed into each plate. To meet expected future needs for compound sourcing. The authors developed a single automated system, that fits on a 6 × 10 ft laboratory bench, in collaboration with Sagian, Inc.

The primary commercial hardware consists of an HP ORCA robotic arm on a 3 m track, Tecan 8051 and 5051 diluters, two Zebra 105 bar code printer/applicators, three Symbol bar code readers, two Hamilton syringe pumps and a Mettler AT200 balance. Custom components engineered by Sagian include a microtitre plate elevator, a vial rack elevator and a microtitre plate sealer. Communication is maintained by a Sagian IID; the robot is controlled by HP-MDS and data management is handled by MS-ACCESS.

The essence of the approach was to develop a single system to weigh, dilute and dissolve compounds and then distribute them into microtitre plates with minimal human involvement. Three steps are required to generate the desired microtitre plates of compounds. First, the system individually bar codes empty amber vials and determines the tare weight of each. Second, an approximate amount of compound is transferred manually into each tared vial at a remote log-in station and the identity of each compound is transferred manually in each vial by reading bar codes. Third, the vials containing compounds are returned to the automated system where compound weights are determined, solvent is added and compounds are automatically transferred to 'mother' and 'daughter' plates which are then bar coded and sealed by the system.

The integrated system described will increase the authors' laboratory's capability to provide dissolved compounds for future screens without an increase in the labour involved in compound handling. Although many of the steps involved in compound sourcing can be performed by small workstations, and a few groups have described large integrated systems, the authors believe that they have an interesting and flexible integrated system of modest size that will have an enormous impact on their laboratory's screening programme.

### An integrated laboratory automation system for quantitative high throughput screening (QHTS) with cell-based assays

**Mark E. Goldman, Timothy Walton, Lynn J. Ransone and Carla M. Suto**
*Signal Pharmaceuticals, Inc., San Diego, CA, USA*

During the past decade, large and small pharmaceutical companies have migrated towards centralized high throughput screening operations. One of the many advantages of this approach is that libraries of screening samples can be evaluated rapidly in multiple assays. Combined with the use of sophisticated data management tools, the resulting information can be compared to identify samples (either defined chemicals or natural product extracts) that have selective activity and warrant further characterization. In order to properly compare data across assays, or develop estimates of structure-activity relationships, it is becoming increasingly important to quantify pharmacological responses. To this end, the authors have optimized their robotics and data management systems to conduct quantitative high throughput screening (QHTS).

Compound are prepared using a BenchMate (Zymark Corporation) modified to accept 16 × 100 mm test tubes in the 8 × 12 format. Vials of compounds are placed in boxes (8 × 12 format) and logged into the data base. Using spatulas, approximately 5 mg samples are placed in pre-tared test tubes, reweighed and then automatically solvated (DMSO) to a concentration of 5 mg/ml. The rack of tubes is transferred to a Packard MultiProbe and 500 µl aliquots are placed in deep-well microtitre plates. For the inflammation and osteoporosis targets, cell lines have been developed which are stably transfected with luciferase reporter genes driven by promoters containing appropriate transcription factor binding sites. Screening is conducted using a custom Zymate robotics system (Zymark Corporation). Each week, 2500 individual samples are tested in six assays (15 000 sample-assays/week). This represents a six-fold increase throughput compared to the semi-automated workstation assay. In addition, the Zymate System uses one quarter of the personnel required for the semi-automated assay. The quantity of data generated by the Zymate System has exceeded the laboratory's ability to use Microsoft Excel spreadsheets for data reduction and analysis. An Oracle-based client–server data management system is used to register compounds (synthesized at Signal as well as those purchased from commercial sources) and to reduce/compare data from many assays. This QHTS system has allowed the authors to significantly increase productivity, reduce screening personnel and accurately compare results with greater confidence.

### Development and implementation of microplate immunoassays employing Zymark robotics

**J. Patrick McCurley and Rose E. Sekulovich**
*Chiron Biocine, Department of Analytical Viral Immunobiology, Emeryville, CA, USA*

The authors' group is responsible for the development and implementation of assays to support Chiron Biocine clinical vaccine trials. With a rapidly expanding vaccine programme, and some projects already in phase 111 trials, standard manual assays were not adequate for the required throughput. Therefore several automation steps have been implemented to facilitate the receiving, tracking, assaying, data processing and data reporting process for multiple samples.

Samples labeled with bar codes generated in-house are compared with corresponding labels on the packing list. The software program identifies discrepancies in the shipment, assigns sample storage locations and records relevant shipment information (date of shipment, shipper and receiver IDs, etc.). A report of storage locations can be generated by entering the sample IDs manually or from a file. Assays on selected samples are then run in a fully automated system. A Zymark PyTechnology system has been adapted to perform microplate immunoassays, and two robots are used full time to run eight different ELISA format assays.

The data processing software matches the sample ID to the data, generates a graphical analysis and analyses the results obtained for the standards, controls and samples to determine if the acceptance criteria have been satisfied. Preliminary results are stored in a table for review by the technician; after the review, results are electronically transferred to a final database for reporting.

Finally, the authors are evaluating the feasibility of adapting a Zymark robot for microplate-based virus neutralization assays, to increase throughput and reproducibility relative to conventional plaque assays.

### MANAGING LABORATORY AUTOMATION

### Managing a high throughput screening group within an international organization

**Carol Ann Homon**
*Boehringer Ingelheim Pharmaceuticals, Inc., Ridgefield, CT, USA*

During the last decade, the pharmaceutical industry has developed high throughput screening (GTS) into a major tool for the discovery of such drugs as Paclitaxel (TAXOL® product), and Tacrolimus (FK506). At Boehringer Ingelheim Pharmaceuticals, Inc. (BIPI), the HTS programme identified compounds which resulted in the discovery and development of nevirapine and ontazolast. The probability of finding new structural leads increases with the number of compounds screened. Hence, successful screening requires a high throughput of compounds. HTS within an international company must be organized on a world-wide basis. BIPI is part of the Boehringer Ingelheim companies, a large international pharmaceutical firm. Recently, Boehringer Ingelheim has organized three dispensary units at Ridgefield, USA; Ingelheim, Germany; and Biberach, Germany. The entire Boehringer Ingelheim library is available for the HTS programme through these three dispensaries. Additionally, natural products and peptide libraries are also screened, as are compounds obtained from outside sources. Screening centres for Boehringer Ingelheim have been established in Ridgefield, USA, Biberach, Germany, and Vienna, Austria. The activities of the dispensaries and the screening centres are co-ordinated by an international group within Boehringer Ingelheim. This is responsible for all logistical and technical aspects of the HTS programme.

### Managing automation for high throughput screening

**Derek J. Hook**
*Bristol-Myers Squibb, Biomolecular Screening, Wallingford, CT, USA*

Over the last decade the pressure on new drug discovery in the pharmaceutical industry has placed increased emphasis on high throughput screening of chemical compound libraries, fermentation extracts, plant samples and now combinatorial libraries as a source of new chemotypes for novel targets.

In order to accomplish this goal, automation has become pivotal in achieving the necessary productivity in laboratories to meet challenges. The key factors critical to implementing automation in pharmaceutical laboratories are closely related to those elements for analytical laboratories. There are, however, special needs for screening, and the growth of recent symposia and courses on high throughput drug discovery show how important automation is to large pharmaceutical houses and smaller biotechnology companies.

The experience of the pharmaceutical industry in managing the introduction of this automation shows that there are common factors that have been experienced by many companies. Novel technologies are driving the emergence of new types of instrumentation in the vendor laboratories and show some of the possible directions that might be taken by research laboratories in the pharmaceutical industry. These directions will continue to put strain on older automation concepts and they will continue to challenge the boundaries of robotics and automation capability.

### Enabling biotechnology research using laboratory automation

**Steven D. Hamilton, Jason W. Armstrong, Richard A. Stanton and James V. Petersen**
*Amgen Inc., Thousand Oaks, CA, USA*

For many years, analytical laboratories have realized increased productivity through the use of laboratory automation. Similar technology is now becoming an important tool to enable biotechnology and pharmaceutical discovery research to progress more quickly and efficiently. More importantly, automation enables researchers to develop discovery processes that go well beyond the human capability to manipulate, track and analyse.

Automation in the research environment involves such technologies as robotics, automatic identification, machine vision, informatics, and image and signal processing. User friendly, robust and flexible automation must be developed and/or applied in an environment where project speed is critical and research directions change rapidly. These needs have led to the creation of the Amgen Research Automation Technologies group, whose focus is to span the boundary between automation development and research biology and chemistry.

The motivations, problems, rewards and strategy related to implementing automation within a biotechnology research organization were discussed, using examples of the Amgen automation programme including: automated colony imaging and picking; high density spotting, analysis and re-arraying; automatic identification (bar coding) and tracking; high throughput PCR preparation and thermal cycling; automated plasmid preparations; and high throughput compound screening.

### CHEMICAL ANALYSIS

### The role of a sample preparation robotics system at a corporate analytical research and development laboratory

**Matthew Benning**
*International Specialty Products, Inc., Wayne, NJ, USA*

International Specialty Products (ISP) is a manufacturer of high performance specialty chemicals. The products have diverse applications as ingredients or processing aids in personal care, pharmaceuticals, agricultural and beverage formulations. The diversity of product applications is equally matched by their chemical morphological characteristics. Products are available as insoluble powders, soluble powders, highly viscous liquids, and non-viscous liquids.

The ISP HPLC method development group is responsible for methods development and validation in support of research, marketing, quality assurance, and manufacturing. The corporate effort on continuous product improvement has placed demands on ISP's laboratory that challenge current manpower capability. Routine analysis in support of research projects and product lines prior to HPLC method implementation and validation at manufacturing facilities, are a significant portion of the laboratory's workload.

An application has been developed to allow robotic sample preparation for a variety of products, matrices and procedures thus freeing the chemists to pursue HPLC method development related activities including instrument setup and data processing. A Zymate II robot with a System V controller was assembled from standard pysections. Subsequently, hardware and software modifications were implemented and validated to enable the system to fit into our highly automated laboratory.

The uniqueness of the robotic system described is in the software design that allows a variety of samples and tasks to be performed by users with different sample preparation requirements. The success of the system is measured by the increased routine workload handled by a small group with increased productivity. Finally, the robotics system is one part of an automated laboratory. Integration as evidenced by data portability, integrity, and some regulatory concerns is easily met and enhanced with this implementation.

### Automated procedures for weighing small amounts of samples

**H. Speck, M. Kranz, M. Luebke, W. Schmid and E. Sametschek**
*BASF AG, Ludwigshafen, Germany*

Sample preparation for ICP-spectrometry consists of two steps: weighing and disclosure. To automate the first step, the authors designed a laboratory robot (in co-operation with Zymark), which can do the following tasks:

(1) Identify samples by reading barcode and getting information on the samples from a LIMS.
(2) Open and close different capped (crimp caps, screw caps, snap caps) and different size sample vessels.
(3) Weigh liquid and solid samples in amounts of 100–500 mg into different target vessels.
(4) Use different tecnniques for weighing in solid samples.
(5) Transfer the weight data to a LIMS.
(6) Label the target vessels with barcode and letters by using an ink jet technique.

The main challenges proved to be handling of a large variety of sample vessels, finding adequate and robust techniques for weighing solids of varying consistency in small amounts, and integrating the robot system into an existing on-line data collection system and a LIMS.

### Automating the vapour test method for evaluating chemical protective fabrics

**Raymond E. Andreotti, Cyrus Kendrick, and Donald Rivin**
*U.S. Army Natick Research, Development and Engineering Center, Natick, MA, USA*

An automated vapour test method that provides a rapid and safe means of evaluating the adsorptive capacity of fabrics used in chemical protective garments has been developed utilizing laboratory robotics in conjunction with an automated effluent detector. Sequential vapour challenge analysis of fabric samples can be performed in a pneumatically operated computer-controlled test cell that was designed and developed not only to facilitate the sample loading and unloading procedure, but also to provide a test environment for obtaining precise and reliable adsorption kinetics. A laboratory robot was programmed to replace the repetitious and tedious tasks formerly performed manually in the vapour test. A personal computer is interfaced to the system and is used for programming and control of the robotics as well as collecting, calculating, and storing effluent concentration test data output from the automated detector. Operational programs were developed that can easily be updated to accommodate changing protocols, such as varying chemical concentrations, flow velocities, temperature, and exposure times. Use of this type of technology for defence- and environment-related applications would ensure that safe and reliable testing could be conducted in governmental and industrial settings.

### A complete custom automation of a commercial analytical instrument with a Zymate® PyTechnology™ laboratory robot system and the associated computer interface

**Mitchell E. Meyer, Collin B. Hitchcock, H. Robert Pinnick, Jr. and Gordon R. Stallings**
*Phillips Petroleum Company, Research & Development, Bartlesville, OK, USA*

The challenge of completely automating an analytical technique is often related to the interface between the robot, the analytical instrument instrument, a Laboratory Information Management System (LIMS), and the associated sharing of data between these systems. This paper discussed an approach employed on three Applied

Research Laboratories (ARL) Inductively Coupled Plasmas (ICPs) to fully automate these analytical instruments utilizing a customized Zymate® PyTechnology™ system, a desktop computer (PC), a LIMS system, and a local area network (LAN). This design can be used for future robotization and automation efforts with other commercially available instrumentation.

### Combining robotics with UV-VIS-NIR spectroscopy

**Carl G. Zimba**
*Polaroid Corporation, Cambridge, MA, USA*

In the development of new photographic media it is necessary to evaluate the colorimetry of a large number of dyes and other components. As these prototype materials are synthesized, the absorption of these materials in the ultraviolet, visible, and near-infrared spectral regions are measured to verify both their absorbance maxima and their absorption intensity.

As part of an ongoing effort to automate routine analytical spectroscopic measurements, a laboratory robot has been combined with both a UV-VIS and VIS-NIR spectrophotometer. The result has been the creation of a highly automated instrument which prepares solutions from powder or crystalline samples using a variety of solvents, and measures the absorption spectrum.

Using this instrument, sample characterizations can be accomplished with more rapid turn-around, more precision and accuracy, and in some cases with less material. Furthermore, the available staff can be better utilized to meet the needs of Polaroid's analysis clients.

The presentation gave an overview of both the hardware and the software of this automated spectrometer and compared the performance of this instrument to manual methods. Potential applications to a more traditional QA/QC environment were also discussed.

### Automation of a NMR measurement of finish-on-fibre

**James Rodgers and Joel Weekley**
*Fibers Division of Monsanto, Gonzalez, FL, USA*

Finish is applied to the fibre surface primarily to assist the fibre's performance during downstream processing. The quantity of finish applied to the fibre surface is called Finish-On-Fibre (FOF). FOF is an important process and quality control parameter for textile fibres and yarns, and it has historically been measured by solvent extraction techniques. A Nuclear Magnetic Resonance (NMR) method, using the bench-top Oxford QP20 pulsed NMR analyser, was developed for the measurement of FOF on nylon fibre products (carpet, tire, conductive fibre). The NMR instrumentation yielded rapid, cost effective, accurate, and precise non-destructive measurements of FOF. Since the NMR analysis requires no solvents, significant environmental and cost-related benefits were realized. Excellent FOF agreement was obtained between the NMR method and a standard solvent extraction technique. A program was initiated to interface the NMR unit to a laboratory automation system. Several automation vendors were compared using a Comparison Matrix (technical capabilities, partnership potential, economics). A Zymark Zymate XP system was determined to be the optimum laboratory automation system for this application. A joint Monsanto-Oxford-Zymark program was established to implement hardware and software integration of the Zymate XP system to the Oxford NMR unit and to Monsanto's QC and LIMS systems. Customized Oxford-Zymark software allowed control of the NMR and robotic functions by the System V controller. Customized Monsanto-Zymark software resulted in the successful integration of the NMR results into the Plant LIMS system.

### Adsorbable organic halogen compounds (AOX): robot-based automated analysis

**Helmut Dillenburg and Bernhard Koerner**
*Solvay Alkali GmbH, Rheinberg, Germany*

The parameter AOX (DIN 38409 T14 Adsorbable Organic Halogen Compounds, where X = Cl, Br, J) is, due to legal regulations and prescriptions, one of the most 'popular' analytical parameters in Germany, if not in Western Europe, for the analysis of water and wastewater. But it is also very time-consuming and therefore costly. A well-trained laboratory operator can analyse about 8 to 10 samples (double estimation) per shift. Thus a lot of attempts have been made to automate all of the analytical procedure or at least major parts of it.

The principle of measurement is as follows: the organic components of the sample are adsorbed on active carbon. Interferences of inorganic halogen compounds are removed by rinsing the adsorption-columns with an aqueous solution of sodium nitrate. The loaded active carbon is then combusted in an oxygen stream. The generated HX are absorbed and the mass of halogen is detected.

This paper described a fully automated, Zymate-robot-based, DIN-compatible procedure, comprising sample identification, enrichment step, sample transfer and interfacing to a commercially available automated analysis system, data collection, evaluation and documentation of the results. Starting with the validation process, results for calibration standards and real-life samples are compared with the results obtained by the manual DIN-procedure in parallel. The comparison showed excellent consistency. The sample capacity of the system is 10 samples per shift (triple estimation and calibration) and can be easily adapted to specific needs.

### DRUGS IN BIOFLUIDS

### An evaluation of automated solid-phase extraction systems

**R. Eric Schmidt, L. A. Kosobud, N. S. Khoshaba, and K. J. Miller**
*G.D. Searle & Company, Skokie, IL, USA*

An evaluation of commercially available automated solid-phase extraction (SPE) systems to perform the extraction of pharmaceuticals from plasma and urine samples was conducted. The evaluation focused on increased throughput and the creation of automated assays which could be transferred to contract laboratories for routine analyses. The Gilson ASPEC XL™, Hamilton MicroLab 2200™, Zymark BenchMate™, Zymark RapidTrace™, and the Cardinal AutoChem Workstation™, were evaluated. The evaluation focused on three components: (1) hardware, (2) software/programming, and (3) application of an established assay presently using a Zymark PyTechnology robot. For the hardware and software/programming, the advantages and the disadvantages of each system were generated. For the application, the analysis time, carry-over, precision and accuracy were compared and conclusions were drawn regarding system applicability for automated sample preparation.

### Use of a Zymate system for bioanalytical automation as a workstation

**Michael Wirth, Mats Svensson and Torbjorn Arvidsson**
*Pharmaceutical and Analytical R&D, Analytical Chemistry, Astra Pain Control AB, 151 85 Sodertalje, Sweden*

Robotics in bioanalytical work often comprises fully automated systems developed for a given method. This paper introduced a concept based on automation of the bioanalytical work using different types of general workstations. Several such stations are commercially available—dilutors, solid-phase extraction systems, autoinjectors etc.

A new type of workstation using a Zymate robotic system has been developed for the necessary steps following extraction (evaporation, reconstitution and transfer to autosampler vials including capsulation). The development, test and validation of the robotic system was discussed. The concept has been applied in a bioanalytical method for determination of local anaesthetic drugs in blood plasma using liquid–liquid extraction and gas chromatography.

### Flexible automation tools for drug metabolism

**Thomas L. Lloyd and Michael G. Dodds**
*Drug Metabolism Department, Glaxo Wellcome, Research Triangle Park, NC, USA*

Robotics was first introduced into most drug metabolism laboratories as a means to automate routine assays. Existing methods for biological fluid sample analysis were transferred to an automated platform to support large clinical studies. Systems were often dedicated to one application and the decision for automating a project hinged on whether the workload for that project justified the investment in equipment and set-up time.

As the flexibility and variety of automation tools progressed, the range of application extended upstream in the drug metabolism process. Robotic systems are still used to automate analysis for large clinical studies. However, they are also used for smaller volume pre-clinical and research support applications. The automated systems are flexible enough to handle up to six different assays in a week and multiple compounds within an analytical run. Bioanalytical methods are developed and refined directly on an automated platform in conjunction with experimental design software. By minimizing or eliminating set-up time between applications, a steady supply of low volume bioanalytical applications can also be cost effective.

The breadth of automation for these applications has expanded as well. Bioanalytical sample preparation techniques include solid phase extraction, liquid–liquid extraction, protein precipitation and dialysis. Sample container labels can be generated and applied automatically for a variety of different container dimensions. Standards and quality controls can be prepared from a concentrated stock and stored in the same labeled containers. A number of sample preparation processing options are available. Prepared samples can be analysed on-line or stored in autosampler vials. Automation continues to be developed for new diversified applications within drug metabolism.

### Extraction of morphine and codeine from urine samples using the Zymark RapidTrace

**Francis X. Diamond, William H. Vickery and John de Kanel**
*National Medical Services, Inc., Willow Grove, PA, USA*

Morphine and codeine are the two opiate drugs designated by SAMHSA (Substance Abuse Mental Health Service Administration, formerly the National Institute for Drug Abuse, NIDA) for analysis in employment and pre-employment situations to determine if heroin, codeine or morphine have been used. Numerous methods have been published describing methods to extract morphine and codeine from urine either by traditional liquid/liquid means or by solid phase extraction. Liquid/liquid extractions are time consuming, difficult to automate and require great analyst skill if they are to produce reproducible and accurate results. Each manufacturer of solid phase extraction cartridges has published methods to accomplish the extraction of morphine and codeine from urine. For the purposes of evaluating the Zymark RapidTrace™ automated extraction workstation the authors chosen 3 ml Confirm HCX Isolute™ cartridges which are available in the USA from Jones Chromatography, Inc. The method was programmed into the RapidTrace™ software without modification. A set of 10 Zymark RapidTrace sample preparation units controlled by one personal computer was capable of extracting codeine and/or morphine from 50 urine samples per hour. The samples were then evaporated to dryness at 40°C using a Zymark TurboVap. This step took approximately 10 minutes. The samples were then derivatized by reaction with 50 μl (BSTFA with 1% TMCS) in 501 μl ethyl acetate for 30 minutes at 70°C in a sealed tube. The samples were then transferred to autosampler vials and analysed by GC/MS. A technician would previously have spent at least three hours to prepare these extracts manually—the use of the RapidTrace frees the technician to work on more productive things such as data review for two of the three hours.

Two millilitre urine samples known to be negative for codeine and morphine were spiked with deuterated codeine and morphine at levels of 50 ng/ml as internal standards and with codeine and morphine at varying levels to evaluate the recovery, linearity and carry-over of the extraction method when performed by the Zymark RapidTrace. The results for codeine and morphine are shown in tables 1 and 2. The standards at 150, 300, 600, 1200 and 2400 ng/ml were used to determine back calculated levels for these standards and to quantitate the controls at 240 and 360 ng/ml, as well as levels in various blank samples. Urine samples were extracted through the procedure after spiking it with 50 000 ng/ml of morphine or 100 000 ng/ml codeine. Analysis of the blank samples following these spiked samples was done to estimate carry-over. The procedure used was identical to the routine procedure which would be followed for real samples. No extra manipulative steps were added in an effort to minimize carry-over. The extracts from the 50 000 ng/ml or 100 000 ng/ml samples were not derivatized nor analysed by GC/MS since potential carry-over from the GC autosampler could have confused the results.

Under SAMHSA guidelines, an affirmative cut-off of 300 ng/ml is used to administratively differentiate positive samples from negative ones. Irrespective of other data, samples containing codeine or morphine below this administrative level are reported to clients as negative. Direct quantitation of the blanks following the 100 000 ng/ml codeine spike yields 65 ng/ml or 0·07% carry-over.

*Table 1. Codeine analysis: Diamond et al.*

| Spike ng/ml | Calculate D level, ng/ml | Ion ratio 229/371 | Ion ratio 356/371 | Ion ratio 229/356 |
|---|---|---|---|---|
| 150 | 160 | 0·32 | 0·13 | 2·5 |
| 300 | 340 | 0·37 | 0·12 | 3·1 |
| 600 | 640 | 0·45 | 0·13 | 3·5 |
| 1200 | 1100 | 0·59 | 0·14 | 4·2 |
| 240 control | 220 | 0·38 | 0·13 | 2·9 |
| 360 control | 320 | 0·40 | 0·12 | 3·3 |
| 0 | 0·56 | 2·8 | 1·6 | 1·8 |
| 0* | 65 | 0·39 | 0·15 | 2·6 |

* Blank extracted after the 100 000 ng/ml spiked sample extraction.

*Table 2. Morphine analysis: Diamond et al.*

| Spike ng/ml | Calculate D level, ng/ml | Ion ratio 401/429 | Ion ratio 414/429 | Ion ratio 401/414 |
|---|---|---|---|---|
| 150 | 150 | 0·28 | 0·43 | 0·65 |
| 300 | 330 | 0·30 | 0·46 | 0·65 |
| 600 | 560 | 0·29 | 0·45 | 0·64 |
| 1200 | 900 | 0·31 | 0·47 | 0·66 |
| 240 control | 210 | 0·32 | 0·53 | 0·60 |
| 360 control | 330 | 0·33 | 0·47 | 0·70 |
| 0 | 1·1 | 0·51 | 2·3 | 0·22 |
| 0* | 16 | 0·34 | 0·54 | 0·63 |

* Blank extracted after the 50 000 ng/ml spiked sample extraction.

For morphine, the blank following the extraction of the 50 000 ng/ml spike yielded 16 ng/ml or 0·03% carry-over. Some of this level is derived from the background noise. The blanks extracted after relatively low levels of benzoylecgonine quantitate to approximately 1 ng/ml, for example. These levels are all far below the administrative cut off of 300 ng/ml.

### Fully automatic determination of the enantiomers of amlodipine in human plasma by robotic sample preparation and gas chromatography

**K. D. Riedel, F. Scharpf, H. Laufen and M. Leitold**
*Department of Pharmacology, Pfizer Mack, Illertissen, Germany*

A sensitive and specific gas chromatographic (GC) method using a Zymark II robotic system for complete sample preparation was developed for the determination in plasma of the enantiomers of amlodipine, a calcium channel blocking therapeutic agent. Plasma samples were alkalinized and extracted with tert.-butyl methyl ether (TBME). Amlodipine and the internal standard (a Cl analogue of amlodipine) were then back extracted into citric acid. After discarding the organic layer the aqueous layer was alkalinized and again extracted with TBME. The ether phase was then evaporated to dryness under a stream of nitrogen. For chiral derivatization the dried extracts were reconstituted into a solution of (+)-(S)-α-methoxy-α-trifluoromethylphenylacetyl chloride. After removing the excess reagent with potassium carbonate the diastereoisomers were then analysed by GC with electron capture detection.

The limit of detection of this method is 0·02 ng/ml plasma for both enantiomers. The day-to-day %CV ranged from 2·6 to 11·7 (both enantiomers). The corresponding within day %CV ranged from 2·6 to 11·7. The Zymate system performs the complete extraction, back extraction, evaporation, derivatization and transfer of the extracts into autosampler vials for gas chromatography. The system consists of the following components (number of modules in brackets): dual-function hand (2), liquid-transfer hand (1), power and event controller (2), rotating overhead shaker (1), centrifuge (1), evaporator (1), screw capper (1), crimp capper (1), vortex mixer (1), dispensing station (2), pneumatic dosing station (1), as well as various sample racks.

A total of 50 samples can be automatically prepared by the robot within 30 hours. This corresponds to a total sample throughput of about one sample per 35 min, which is similar to the chromatographic time.

### PHARMACEUTICAL ANALYSIS

### Analysis of water soluble vitamins in multivitamin-mineral supplements on an automated tablet processing-liquid chromatographic system

**Andrew L. Deputy and Lionel P. Murray**
*Bayer Corporation, Consumer Care Division, Elkhart, IN, USA*

This presentation described a project to develop a rugged and reliable automated analysis system for ascorbic acid, folic acid, niacinamide, panthothenic acid, pyridoxine, riboflavin and thiamine in multivitamin-mineral supplements. In addition, the analytical system must be robust enough to process a large number of samples with varying formulations and physical characteristics. These differences in products can include colour, flavour, coating, tablet shape, tablet hardness, types and amounts of active ingredients, and multiple tablet excipients.

In order to produce such an automated system, a chromatographic separation was developed which is extremely rugged and insensitive to minor changes in the tablet excipients, including the flavours and colours. During the development of such a rugged separation, the chromatographic separation was optimized for capacity factor ($k'$) and selectivity ($\alpha$). Because of the interrelated nature of these two parameters, an 'expert' approach was used for the development of the HPLC separation. In addition, post-column derivatization was employed for those vitamins which either do not have an easily accessible chromophore or whose chromatographic behaviour does not yield the desired selectivity over interfering components of the table matrix. For the multivitamin-mineral supplements, ascorbic acid and pantothenic acid meet these criteria.

The optimization of the aqueous extraction of the water soluble vitamins was accomplished using a Taguchi Methods® Optimization scheme ($L_9$) to examine the influence of various parameters on the extraction of the vitamins from the tablet matrix. Extraction parameters examined included homogenization time, extraction temperature, extraction solvent pH, and pre-extraction soak time. Parameter conditions and ranges were selected to optimize the precision ($S/N$) of the extraction process; therefore, providing a rugged automated anlaysis system. In summary, a rugged, precise analysis system is necessary for use in the routine release testing of pharmaceutical products. By the use of experimental design techniques, it is possible to build ruggedness into the analytical system during development.

### The BenchMate II TPW—from installation to financial success

**Simon Smith**
*SmithKline Beecham Pharmaceuticals, Manor Royal, Crawley, West Sussex, UK*

The Quality Assurance Department at SmithKline Beecham performs the testing of samples for stability, process development/new product introduction and routine release for sale. As a consequence of long-term management objectives, the goals are to reduce laboratory testing time to 24 hours and eliminate waste while maintaining or reducing costs. Within the analytical laboratory, automation can provide the key to these business challenges. If careful implementation is employed, the automated laboratory will emerge as the successful, cost effective and efficient laboratory of the future. This result will consequently give that certain leading edge to any product in the aggressive market place and allow greater flexibility as the automated laboratory will be situated at the end of the production line.

The release for sale and stability testing of a particular film coated tablet has been automated within the laboratory by the successful implementation of a BenchMate II Tablet Processing Workstation. Initial justification for the purchase of the system was based on the view that it would replace an analyst and create extra capacity. This view has been realized but many hidden benefits have emerged, with the main ones being a reduction in inventory costs and the number of samples requiring retesting.

Due to the success of this first automation challenge, a second system was purchased, validated and implemented in a third of the time of the first system and is now also used for the testing of a second product.

### Robotic-LC analysis of Benadryl tablets

**Fern Smith and Harold Shaw**
*Parke-Davis/Warner Lambert Company, Brockville, Ontario, Canada*

The BenchMate TPW Workstation (Zymark Corporation, Hopkinton) consists of two parts, the BenchMate Workstation and the Tablet Processing Workstation. The BenchMate TPW Workstation provides a versatile, cost effective alternative to manual sample preparation for content uniformity assays, potency (composite) assays, and dissolution testing. These routine tests require samples to be ground, dissolved, filtered, diluted, and analysed by chromatographic techniques. The BenchMate TPW Workstation utilizes computer controlled wet-grinding homogenization, a liquid management system, an internal four place analytical balance, and a three place top loading balance to consistently provide accurate and precise results. The workstation also provides an audit trail for all sample weights used and sample volumes transferred.

### Fast dissolution sampling and analysis using a Zymark dissolution robot and on-line UV/visible spectroscopy

**Daniel W. Barrow**
*Bristol-Myers Squibb, New Brunswick, NJ, USA*

Dissolution testing is a common analytical procedure used throughout the pharmaceutical industry to meet legal requirements for compendial drugs and to provide a quality control measure for solid dose forms in manufacturing and research operations. Fast on-line UV/visible dissolution sampling and analysis have been achieved using a customized Zymark dissolution robotic system. The system is capable of sampling and analysing solutions from a single vessel in less than 50 seconds: allowing six vessels to be tested in under five minutes. It is equipped with a second six vessel dissolution tester thus enabling analysis of 12 dissolution samples in under 10 minutes. The dissolution procedure is fully automated including analytical standard analysis, system preparation, sample addition and analysis, system clean-up, results calculation and print-out. Common productivity gains range from 50% when compared to conventional robotic dissolution systems to 300% when compared to manual methods of analysis.

This robotic system has been used in the analysis of several pharmaceutical products. The presentation showed system schematics and system validation data.

### Using a PyRobotic system to update and automate the existing analytical methodology in a currently marketed product

**Allan L. Greenberg and Phillip A. Lane**
*Analytical Research and Development, The R.W. Johnson Pharmaceutical Research Institute, Raritan, NJ, USA*

Laboratory robotics have been previously demonstrated to be most efficiently used where conditions make reproducibility important and sample throughput prime considerations. Most of the previous implementations in the authors' laboratories have been in the application of robotics to new dosage form sample preparation schemes. However, in the pharmaceutical industry, especially in today's climate of having to work faster and more productively, it is easy to go backwards and implement automation into pre-existing analytical procedures rather than only considering application of robotics to new chemical entities.

The robotics programs used were taken from a cream application, which was presented two years ago at ISLAR. A comparison of the flow diagrams of the original program and the desired program was used in the presentation to indicate what part of the program could possibly be salvaged. The programs were modified to meet current analytical requirements. The new robotic procedures also incorporated a system suitability check and preparation of standards.

The results from the assay of robotically prepared cream samples were compared to manually prepared samples. The evaluation concluded that the results were statistically equivalent. The assay reproducibility and sample throughput was presented for both cases.

### Using an expert system with laboratory robots to provide flexibility in the analytical development laboratory

**M. E. Hinshaw and D. A. Jackson**
*Lilly Research Laboratories, Eli Lilly and Company, Indianapolis, IN, USA*

Until recently, robots have been of limited use in the authors' pharmaceutical development laboratories. The time required to plan, build, and validate systems is not well accommodated in today's accelerated development environment and once the bolus of samples is through the system, the robots are generally not flexible enough to be used for other projects without substantial modification.

This problem has been addressed by changing the basic approach to building robotic systems at Eli Lilly. Each system is now constructed to allow for multiple sample preparation techniques using various container sizes, regardless of the requirements of the immediate application. Instead of programming for specific applications, an expert program interacts with the user to create the application program. The installation qualification and operational qualification protocols were used to demonstrate that the robotic system hardware and expert program are working as intended. The performance protocols were used to validate the application programs generated by the expert program and to demonstrate equivalence to either manual or other automated procedures.

### Installation, validation, and implementation of the Zymark MultiDose Automated Dissolution Workstation for profile testing of tablets using the paddle apparatus

**John J. Mullen and Timothy J. McCormick**
*The DuPont Merck Pharmaceutical Company, Wilmington, DE, USA*

The MultiDose Automated Dissolution Workstation automates the paddle method for dissolution testing of pharmaceutical solid dosage forms. With minimal operator set-up the system will automatically dispense dissolution media, check the temperature, introduce tablets, and provide selected profile sampling at intervals as close as five minutes. The installation process supplied by the manufacturer helped provide for a problem-free start up. Zymark engineers were able to set-up and have the system operating in several days.

A thorough system hardware and software validation was conducted which included the IQ, OQ, and PQ of the system. The Installation Qualification confirmed that the analytical balance, thermisters, pumps, and all other components were installed correctly and that they operated as expected. The Operation Qualification verified the functional and applicational performance of the workstation. All components did operate as they were intended throughout anticipated ranges. The Performance Qualification of the system was the actual execution of an analytical procedure without an error and in the correct sequence.

Product/application specific validation was also conducted which included: calibration, sample solution carry-over checks, vessel washing effectiveness and manual versus automated comparisons.

The results of the above installation/validation process were presented along with the implementation of the system for product stability testing.

### Automated dissolution testing in the validation of tablet coating procedures

**Kevin K. Olsen**
*ESI Lederle Generics, Pearl River, NY, USA*

A common medication is manufactured in four strengths by ESI Lederle Generics at their Pearl River New York facility. Validating a recent change in the tablet coating operation required extensive dissolution testing in which the tablets are allowed to dissolve under tightly controlled conditions and the concentration of the active ingredient is measured as a function of time. Such testing is very commonly used to monitor the release characteristics of a wide variety of pharmaceutical preparations.

Since two separate pieces of coating machinery were involved, almost all the analyses were done twice. Each of the four tablet strengths required testing on uncoated tablets, dissolution profiles, and testing of tablets taken from different steps in the coating process. The estimated time to complete the testing by manual methods was over 300 hours. In addition, the laboratory had to support its normal workload of quality control analyses.

Substantial savings of time were achieved by automating the testing procedures using a Zymark MultiDose, an autosampler on the UV/Vis spectrophotometer and a Beckman LIMS to manage data handling and routine calculations. The Zymark MultiDose was the critical element because it allowed the entire test to be automated, including the very labour intensive steps of cleaning the apparatus and preparing it for the next sample. The final result was a documented saving of slightly more than 50% in analyst time. Results obtained by the automated system were in good agreement with those from manually prepared samples.

### DATA HANDLING

### Automation of data handling for trace analysis of drug active in on-line cleaning validation samples

**Jon P. Sadowitz and Thomas D. Groeschner**
*Schein Pharmaceutical Inc., Carmel, NY, USA*

In response to current concerns a cleaning validation program was developed. Within this program, it became necessary to automate the analysis of data to increase productivity within a small group solely responsible for the analysis of all cleaning validation samples. Until very recently, data analyses were performed by hand, which consumed much of the analyst's time when dealing with the large number of samples associated with a cleaning validation study: a small cleaning validation study of 50 tubes would require over 150 individual calculations. For a small staff of two chemists, automated analysis was essential.

This presentation included the development and overview of the final product utilized in the automated transfer of raw chromatographic data to transform these data into a workable format to interpret the results of a cleaning validation study.

### LABORATORY WORKSTATIONS

### Automating the process of removing coating materials from tablet formulations

**Kathy Duquette**
*Wyeth-Ayerst, Rouses Point, NY, USA*
**and Chris Werner**
*Bohdan Automation, Mundelein, IL, USA*

This presentation demonstrated an automated system that is capable of removing the coating material from the active inner core of pharmaceutical tablet formulations. In addition to selectively removing the coating material, the instrument also calculates and records the average tablet core weight.

The initial intention of the automated workstation was to free the analyst from the time-consuming task of washing the tablets by hand. The analyst is now able to wash up to 12 batches of coated tablets with a very short amount of time needed to set up the machine. In addition to improving the use of valuable employees, more consistent results are achieved using the automated approach.

The tablet washer has the capability of washing, rinsing and drying the tablets. A variety of 'run parameters' are addressed by the analyst at the beginning of the program. The computer program also stores the weights of the tablets in its memory, as well as a hard copy print-out. This allows the laboratory to have both permanent and electronic documentation of the sample preparation and sample data report.

To date, the automated workstation has shown results that are equal to or better than manually prepared samples.

### Automation of a tablet assay using a BenchMate TPW

**Michele E. Lake, David A. Hollowell, James L. Sabatowski and Mark N. Flair**
*Eli Lilly and Company, Lilly Research Laboratories, Indianapolis, IN, USA*

A Zymark BenchMate Tablet Processing Workstation (TPW) has been used to successfully automate a potency determination for tablets resulting in precise results and substantial savings in analyst time. During automation of the potency method, several iterations of the TPW procedures were examined. TPW results generated with the various procedures were compared to manual results (as well as the percentage label claim) until both sets of results were statistically equivalent. Since validation of the automated method, a control sample has been utilized and a control chart of this data has shown the reproducibility of the TPW for the assay of tablets. The TPW data has been analysed to evaluate the method variability (run to run), as well as tablet variability for the established control sample. This presentation described the automated assay and the results.

### Automation of the analysis of an unstable analyte in solid dosage forms with the BenchMate TPW

**L. A. Cavenaghi and E. Gabriele**
*Guppo Lepetit SpA, Anagni, Italy*

With the use of a BenchMate TPW, the authors have been able to automate the analysis of Rifampin in solid dosage forms. This antibiotic, which is widely used for the treatment of difficult infections, is formulated in sugar coated tablets of different strength ranging from 100 to 600 mg, and up to now could not be analysed automatically because the compound oxidizes in solution.

The use of the BenchMate TPW with its capability of preparing standards and samples (weigh, dissolve, dilute and inject) at the appropriate moment has solved the problem.

A new method has been developed to cover all those in use for the different formulations. The method covers the range 80 to 720 mg/sct ($\pm 20\%$ of theoretical content), enabling the laboratory to have an enormous increase in flexibility of response. This increase was achieved with no loss of precision and accuracy of the analytical procedure.

An added bonus of the use of the BenchMate TPW was the ease of the validation with automatic tracking of all the dilutions and thus removing the need to validate the analyses.

### Workstation automation of the biochemical oxygen demand method

**Lars Lindquist, Sonja Soloway and Donna Pocius**
*WMX Environmental Monitoring Laboratories, Inc., Geneva, IL, USA*

A fully automated BOD workstation was developed to automate the Environmental Protection Agency method 405.1. This system has the capability to deliver sample, seed, controls, and dilution water to BOD bottles. The dissolved oxygen is measured with a probe and a water seal is formed when the cap is placed on the BOD bottles.

The operation of the Zymark workstation with a personal computer running Windows™ is very user friendly because it uses Visual Basic images of the samples, racks, and BOD bottles. The user points and clicks on the mouse to screen images of the sample bottles and inputs the information to set up the analyses. The BOD bottles that are analysed are tracked with a bar code reader. The results can either be reported from a standard report or customized by use of other spreadsheet software.

System validation, method detection limits, sample throughput, and performance characteristics were presented.

### VALIDATING LABORATORY AUTOMATION

### The generic dictionary: a new approach to validation

**P. J. Gallant and R. S. White**
*DuPont Merck Pharmaceuticals, Radiopharmaceuticals Division, Process Testing Development, Billerica, MA, USA*

In most GLP/GMP environments, the major downtime to implementing automated systems is the validation process. With respect to the Zymark XP system in DMPC's Process Testing Group, the major drawback to converting the current XP system over to new analyses is validating the new configuration on a relatively unchanged system.

The concept of a generic dictionary places emphasis on the range of functionality versus function for a given application/use. Consider a liquid/liquid transfer station, currently validated and used to aspirate/dispense 2 ml. A generic version of this application would be validated for aspirating/dispensing operations with volumes ranging from 0·5 to 10 ml, as opposed to the fixed volume of 2 ml. When a new sample process is required, any volume between 0·5 and 10 ml could be implemented without revalidating the functioning of the liquid/liquid transfer station.

On a broader scale, the entire dictionary can be designed with this generic focus. The various functions the XP system performs can be broken down into functional units. Functional units are similar to laboratory operation units (LUO), although the emphasis for functional units is on the range of functionality for a given operation versus repetition of that operation.

This presentation outlined the concepts and the authors' experiences in implementing the generic dictionary on an existing XP system.

### Systematic approach to automation validation

**W. J. Ewing and P. J. Gallant**
*DuPont Merck Pharmaceutical Company, Radiopharmaceuticals Division, Billerica, MA, USA*

The trend in today's laboratories is movement toward automation. This advancement is accompanied by the need to validate the automated system. This process can be very long and time-consuming; however, with the proper knowledge and planning, the validation process can be short, painless and very successful. Initially, the idea about an automation project is conceived, and then the research and investigation begins to find the perfect system and design to suit specific needs. A rough layout or system plan should be generated to ensure that the system obtained can perform to the expectations. The functional requirements document should be written in which the system will be described in general and the operational requirements of the system will be stated. A system specification document is also required which is made up of hardware and software design specification of the system.

A validation plan needs to be established that will include the documentation necessary to prove that the system performs its functions properly and is consistent with the functional requirements and system specifications. The validation plan also identifies all the required tasks and responsibilities needed to have a successful validated system.

The actual validation testing can be broken into three parts: installation qualification; operational qualification; and performance qualification. The installation qualification (IQ) test is performed to ensure that all of the equipment is installed according to the manufacturers' specifications and recommendations. The operational qualification (OQ) tests that the system components each perform correctly within their operation ranges. The performance qualification (PQ) provides proof that the system performs all its functions accurately, reliably and in accordance with the functional requirements.

This presentation described the steps involved in validating an automated system and maintaining that system in compliance once it has been validated.

### A generalized performance based approach to the operation qualification of the Zymark BenchMate/ TPW

**David A. Hollowell, Mark N. Flair, Jeffrey D. Hofer, and Michele E. Lake**
*Eli Lilly and Company, Lilly Research Laboratories, Indianapolis, IN, USA*

A generalized approach to the operational qualification of a Zymark BenchMate/TPW has been defined and executed in the Pharmaceutical Analytical Development Division at Eli Lilly and Company. The operational qualification (OQ) is intended to produce documented evidence providing a high degree of assurance that the automated system is capable of reliably performing the desired analytical functions in the laboratory. Using vendor specifications and statistical techniques, rational limits for automated operations have been derived from NIST Class A specifications for the glassware-equivalent manual laboratory operations. This approach was undertaken in order to be able to confidently utilize the equipment for development of analytical methods as well as to validate the automation of manual procedures. This presentation gave the approach, results, and conclusions of this operational qualification.

### DRUG DISCOVERY RESEARCH

### The automation of radioisotopic and luminescence assays for high throughput screening

**Alfred J. Kolb**
*Packard Instrument Company, Meriden, CT, USA*

The number of samples being analysed in high throughput screening has been growing at an acclerating rate over the last five years. To keep up with this demand, pharmaceutical companies have been drawing upon innovations in analytical instruments, microplates, assay technology and robotics. The core instruments in most screening laboratories include liquid handling systems and an array of analytical instruments such as densitometers, luminometers and microplate counters for radioisotopic measurements. These instruments can handle a substantial sample throughput when combined with microplates and assay technologies that reduce, or eliminate, sample processing steps.

Microplates with integral filters have eliminated the need to handle individual filters for the analysis of receptor binding or cell proliferation. The harvesting and analysis of samples occurs in the same microplate, thereby reducing sample preparation time. In-plate assays for immunobinding, receptor binding and nucleic acid hydribidization have been designed that require only a simple aspirate and wash step. Other assay systems completely eliminate the need to separate bound from free label. Many of these assays are available using radioisotopic labels or new luminescent reagents with half-lives of hours instead of minutes. This allows the researcher to choose from a range of methods that best suit the individual laboratory requirements.

The simplification or elimination of separation steps has also simplified the complete robotics automation of screening assays. Filtration assays for receptor binding could not be automated when these assays required the use of filter disks or sheets of filter paper. The development of microfiltration plates and non-separation assays offered a practical solution for robotics automation.

The co-operation of Packard Instrument Company and Zymark is an example of how companies can combine their strengths to offer solutions to the automation of high throughput screening.

### Automated template preparation for high throughput sequencing with BioRobot 9600

**Michael Collasius**
*QIAGEN GmbH, Hilden, Germany*
**and Alex Zrolka**
*QIAGEN Inc., Chatsworth, CA, USA*

The BioRobot 9600 has been designed specifically for high throughput production of ultrapure sequencing templates. Templates can be prepared using either the QIAwell Ultra or QIAprep M13 Systems. The QIAGEN kits have been optimized in our research facilities for use with the BioRobot 9600. The BioRobot 9600 can meet the throughput requirements of any type of laboratory. Depending on the type, source and quantity of DNA templates being purified, the workstation can deliver up to 96 templates in as little as 2 hours.

The BioRobot 9600 hardware can be easily adapted to meet changing research requirements. The modular platform allows the option to upgrade as necessary, or as new technology becomes available. The workstation combines the novel QIAGEN operating system with the Windows user interface to provide eash set-up and execution. The point and click operation can be used to modify existing protocols or even create new applications.

The BioRobot 9600 provides the reliability of proven purification chemistry with the high throughput requirements of modern laboratories.

### An automated system for preparation of radiolabeled antibody

**Jimmy Bruner, Julie Stimmel and Doug Wilson**
*Glaxo Wellcome Inc., Research Triangle Park, NC, USA*

A laboratory robot has been developed to perform the radioligand labeling of clinical quantities of a monoclonal antibody. The system was developed in response to a problem of high radiation exposure to staff using manual manipulations. A small Zymate XP robotic system was assembled to perform radiolabel preparation, radioligand binding and cleanup of the radiolabeled antibody using a spin, size-exclusion column.

A new 0·1–10 μl Eppendorf pipette hand was implemented optimized and characterized. The 0·1–10 μl Eppendorf hand (fabricated by Zymark) uses the variable plunger mechanism of a standard Zymark pipette hand. Custom software was developed to allow variable volume dispensing from the Eppendorf with a linear calibration adjustment made in the software. Accuracy and precision of 5% and

$\pm 5\%$ were achieved at a 1 µl nominal volume with accuracy and precision of 2% and $\pm 1\%$ at 10 µl volume.

The radiation exposure of scientists was reduced significantly, allowing for more frequent and safer experiments. The robotic method improved reproducibility and overall recovery of radiolabeled antibody versus the manual method.

### Motion control of an X-Y stage and autofocus for a microscope using a commercially available vision and instrument control package

**Joe J. Yacobucci, Jeff Guss and Derek Hook**
*Bristol-Myers Squibb, Biomolecular Research, Wallingford, CT, USA*

Many biological responses are accompanied by a shape or fluorescent change. Quantitating these changes by eye using a microscope or a television monitor is tedious and prone to error with the added difficulty of controlling for operator-to-operator variation. The authors report on the application of an X-Y stage and autofocus for a microscope using Ludl motion hardware, and software.

Topics discussed included automated X-Y stages, autofocussing, computer logic commands, image prameters, and the use of a Zymark robot to facilitate unattended operation.

This system has processed unattended over 100 000 microplate wells.

### Automated compound storage and retrieval systems—providing a backbone for high throughput screening programs

**Scott C. Atkin and R. Bruce Jamieson**
*SAGIAN, Inc., Indianapolis, IN, USA*

As High Throughput Screening technologies are implemented in pharmaceutical and agricultural compound discovery processes, the rate limiting step in successful screening programs becomes the ability to handle the 'upstream' preparation of potential compounds. Specifically, as HTS processes become robust within an organization and truly high throughput there comes an ever increasing demand for more novel compounds to screen with improved access to existing libraries. Combinatorial chemistry and acquisition programs can accelerate the identification of new compounds. However, without increasing manpower, the preparation of these compounds can only be accomplished with automated systems.

Automated Storage and Retireval Systems (ASRS) allow for the rapid retrieval of existing compounds for use in HTS programs. A common requirement for many compound management systems is the retrieval of an archive compound from a central storage facility. Subsequent steps may include transferring a sub-sample to another container for distribution to an internal HTS laboratory, transfer and dissolution for shipment and/or short-term storage, creation of 'daughter' microplates for immediate use, storage, or shipment, or other processing steps. Once this intermediate processing is completed, the original source container is typically returned to its original location.

Various strategies exist for automating the storage/retrieval and preparation processes. Approaches include attempting to automate the complete procedure to selectively automating steps in the process. Where full automation has the attraction of reduced manpower requirements, selective automation will normally increase the likelihood of success and potentially results in optimum utilization of manpower with maximum return on investment.

### CUSTOM AUTOMATION

### Custom automation of polyurethane materials testing using visual programming tools

**Steven E. Robbins and Michael E. Rusak**
*Air Products and Chemicals, Inc., Allentown, PA, USA*

Polyurethane foam physical test measurements are fundamental to characterizing end product uses and determine economic feasibility. Visual programming tools were used as the basis for integration of diverse hardware and software necessary in this custom application development. The visual environment improves system operations and minimizes the need for extensive training. This presentation described some of the technical approaches used in automation of data acquisition, instrument and robot control, and system management.

### Integration of laboratory automation for the Human Genome Project

**William Lee, Eric S. Lander, Trevor L. Hawkins**
*Whitehead Institute/MIT, Center for Genome Research, Cambridge, MA, USA*
**and Glynn Searl**
*CRS Robotics Corporation, Burlington, Ontario, Canada*

There is a major need for the development of a system which can accomplish the integrated tasks of DNA isolation and proceed with purification and the set-up of sequencing reactions. The authors demonstrated the feasibility of such a system from both a biochemical and engineering perspective. The authors are collaborating with CRS Robotics Corp., Packard Instruments, Tecan US and Techne Include to design and construct a factory-style laboratory system. The major component of the system is an articulate CRS 255/A robotic arm which is track mounted. The deck of the robot contains several new/ modified XYZ robotic workstations, a novel thermal cycler with automated headed lids, carousels and custom built plate feeders.

Biochemically, the authors employed their solid-phase reversible immobilization (SPRI) technique to isolate and manipulate the DNA throughout the process. The system is flexible and can be modified relatively easily. In this way, one system can be used to accomplish many different biochemical tasks.

### An integrated system for automated radio iodination

**Andy Chang, Greg Bennett and Randy Yen**
*BioAnalytical Technology Department, Genentech, Inc., South San Francisco, CA, USA*

An automated robotics system was developed to perform protein-labeling experiments involving radioisotopes. The reason for integrating this system is to allow people to minimize exposure to radiation, not for high throughput nor for running experiments at a high volume. The system consists of a 1 m HP ORCA arm and four syringe pumps. It is small enough to reside behind appropriate shielding inside a chemical hood from which a charcoal filter traps volatile radioactive molecules. A Visual Basic software program was written to control the robot and to provide a user interface for scientists or technicians. The user can easily change parameters and/or create new methods without having to know programming for the robot. This system addresses safety concerns by reducing human exposure to high levels of radioactivity, offers improved reproducibility and reduces manpower needs.

### The robotic automation of the determination of doses from metered dose inhalers under a wide range of simulated patient usage conditions

**Andrew Monk**
*Department of Robotic Automation, Thurnall PLC, Manchester, UK*

This system described is an automated system providing an repeatable method for handling and firing Metered Dose Inhalers (MDIs) into collection apparatus, measuring loss and determining dosage. The primary elements of the system are: a gantry robot system, shaker/stirrer mechanism, analytical balance, dose collection facility with associated washdown unit, and an autosampler to provide samples for HPLC analysis. The system provides a cost effective and flexible alternative to manual testing.

### Custom engineered automation for scientific research

**D. D. McCampbell, M. F. Fischer and C. E. Ball**
*Midwest Research Institute, Kansas City, MO, USA*

Midwest Research Institute (MRI) has been providing contract scientific research for over 50 years, in fields such as energy, environmental, health, and transportation. The automation group is a multidisciplinary group with backgrounds in mechanical engineering, engineering design, artificial intelligence, computer programming, machine vision, analytical chemistry, and biology.

MRI's specialists have developed robotic systems to increase the throughput and precision of such tasks as natural pesticide screening, oncogene inhibitor screening, biological matrix extraction, and food product analysis. Some of the custom systems developed by the automation group at MRI were discussed in this presentation.

### CHEMICAL ANALYSIS

### Intrinsic viscosity measurement using a laboratory robot

**Philip J. Farrelly**
*Hudson Control Group, Springfield, NJ, USA*

The presentation described the design and development of a robotic system to fully automate the measurement of intrinsic viscosity of polyester materials for a major petrochemical manufacturer. The topics covered included: system design overview; identification of technical challenges; required innovations in hardware and software; software development; operational methods; and future expansion.

### The design, construction, testing and implementation of an automated robotics system

**Juan C. Cadavid and Marie Sabo**
*Clairol, Inc., Stamford, CT, USA*

Keeping in line with today's requirements, the Quality Assurance group at Clairol was faced with the task of doing more with less; i.e. they were confronted with the challenge of making more efficient use of their time. After a careful evaluation of possible alternatives, an automated robotics system was selected to perform the most time intensive analyses in QA's daily workload as well as the ones with the most sample throughput. These analyses were identified to be the determinations of % total alkalinity, % fatty acid, and Brookfield viscosity.

Both the total alkalinity and the fatty acid analyses were implemented using existing analytical autotitrator methods. The existing Brookfield viscosity technique (on a dye/developer mixture) was significantly modified due to physical constraints in automating the method.

The robotics system is capable of handling 130 samples per day with 10 possible combinations of analysis. The possible combinations are determined via bar code scanner from a preprinted list of bar code labels for each sample. This information is sent to the top level user interface program Visual Basic™, an object-oriented programming language which operates from the easy-to-use Windows™ platform. The intelligence and optional help features incorporated into the interface permit operators to obtain results with minimum supervision.

A description of the automated system was presented, along with the requirements posed to the system, the hardware and software utilized, and the system validation schemes.

### ADVANCED TOPICS

### An approach to use low-budget-imaging to automize the detection of liquid–liquid interfaces in labeled test-tubes

**Manfred Jungke, Marius Voehringer, Karlfried Doerr, Christian Lehmann and Jens Luettge**
*Fachhochschule, Frankfurt am Main, Germany*

The main objective of this work was to investigate whether low budget hardware components, combined with a standard PC, could be used to solve a complex task of liquid–liquid extraction in labeled test-tubes. The goal was to detect the separation layer of two transparent colourless liquids, with different refraction coefficients. The tubes may have white labels or light colour labels. The labels may cover up to half of the circumference of the test-tube, they may be machine printed or manually written. There is no need to predetermine the position of the label. The method was tested by using an example of plain water and sunflower oil that had been carefully added before.

One major task was to set up a solution with low cost commercial consumer components such as a black and white camera module, and a consumer PC-video-digitizer, which was used as a frame-grabber. The components were successfully mounted on a Zymate py-plate and were integrated in the automation process under control of a Zymate II System. The method was developed by training electrical engineers in automation tasks in an educational environment.

This work was supported as a research project by the Hessian Ministry of Science and Art.

### Evolution of machine vision enabled automation: from the factory floor to the laboratory bench

**James H. Beyer, Michael E. Dobbs, Paul C. Olsztyn**
*Vision Instruments, Inc., Ann Arbor, MI, USA*
**and Julie Erb**
*Proctor & Gamble Company, Cincinnati, OH, USA*

Historically, the application of machine vision to automation problems has been an expensive, one-of-a-kind effort, requiring special purpose hardware and highly skilled engineers to support an application. The high cost of developing and maintaining a vision enabled automation application restricted its deployment to large volume/high margin manufacturing tasks, such as welding in the automotive industry and circuit board soldering in the computer industry. The increasing computational power of commercial personal computers, coupled with their decreasing cost, has enabled a new machine vision paradigm. PC hosted image processing software can now be combined with robotics and other automation components for a fraction of the cost of systems created just a decade ago. This decreased cost enables machine vision to move from large scale factory automation, to smaller scale laboratory automation. Vision Instruments Inc. has introduced a series of vision enabled products which allow the automation of laboratory processes previously not amenable to automation effort. This presentation examined the evolution of image processing techniques from its DOD roots to its application in the modern laboratory. It also showed the implications of this evolution for the automation of laboratory procedures used in high throughput screening and microbiological counting and classification techniques.

### A modern robotic automation technology for material handling applications

**Karl Sharicz, Ed Fournier, David Feindel and Marc O'Mara**
*Asyst Automation Inc., Wilmington, MA, USA*

Like most manufacturing environments today, the semiconductor industry faces many of the same challenges as those involved with pharmaceutical drug discovery and development. The common denominator in both industries is that traditional methods are no longer sufficient in satisfying the demands for increased yields, reduced costs, and accelerated time-to-market. Comprehensive solutions that essentially redefine the work process and help organizations remain competitive into the next century are required. At the same time, these solutions must offer a total systems approach that allows organizations to retrofit and extend the usefulness of existing facilities, as well as build new cost-effective facilities. In essence, these solutions must provide integrated strategies with a financial and technological pay-off.

Automation, through the use of robotics technology, has made a significant contribution in process optimization and has led organizations toward realizing the goals prescribed by these new demands. For the semiconductor manufacturer, the evolution of more advanced chip designs, larger wafer diameters, safer and more reliable material handling, and needs for reduced particulate contamination have helped create increased opportunities to apply robotic automation.

Since 1981, Asyst Automation engineers have served the needs for the semiconductor manufacturer by pioneering the field of contamination-free robotic material handling systems. These systems enable manufacturers to safely store, retrieve and transport material during the manufacturing process. Materials include bare silicon wafers, wafer containers, bare reticles, and reticle containers. By designing the system to work within its own 'mini-cleanroom' environment, Asyst Automation has helped semiconductor manufacturers reduce space requirements and recover valuable cleanroom floor space.

This presentation, through graphic illustration and videotape, showed robotic material handling in action and demonstrated how the material storage, retrieval, transport, and management process applies in a variety of configurations and applications. These systems provide data links to other aspects of the manufacturing process; the technology is easily learnt and mastered by production personnel through a graphical user interface provided by the OS/2 Presentation Manager control software.

### MANAGING LABORATORY AUTOMATION

### A team approach to the transfer of robotics to a QC environment

**J. R. Tricome, N. Muhammad, S. W. Swieck and J. J. Rice**
*Bristol-Myers Squibb Company, Syracuse, NY, USA*

In today's competitive environment in the pharmaceutical industry and with increased emphasis on productivity and efficiency, a team approach is essential to successful implementation of a robotic system in a QC environment. A QC initiative was undertaken at Bristol-Myers Squibb in Syracuse to implement a robotic system to analyse Penicillin fermentation whole broths on a 24-hour basis. This team effort has resulted in the introduction of robotics to the QC laboratories and a smooth transition to a fully automated analysis technique.

### Approaches to automation for pharmaceutical analysis

**Alan Wickman**
*G. D. Searle, Skokie, IL, USA*

Automation has been an ongoing endeavour within G. D. Searle's analytical group for the past 12 years. Initial successes were with automation of dissolution testing. This was followed by not so successful attempts at automating other sample analysis techniques. With much hard work, lessons learned, and persistence, all major analytical tests have been automated. While automation has meant that analysis capacity has been increased, it has also presented new problems and challenges.

This paper discussed what has been automated at G. D. Searle, what works best and why, the problems, the challenges and a view on what the next five years will bring.

### Regrouping, reforming and re-engineering: applying robotics to new challenges

**Stephen Scypinski, Theodore Sadlowski and John Baiano**
*Analytical Research and Development, Hoffmann-La Roche, Inc., Nutley, NJ, USA*

At the ISLAR meeting over the past several years, many management session and plenary talks have dealt with the reality of 'doing much more with less . . . faster!' Most recently, Dr Phillip Lane of R. W. Johnson PRI spoke of 'The reality of the 90s'. The number of mergers, acquisitions and consolidations in our industry has intensified. All of these actions always result in more work for less people. Hammer and Champy's 1993 book *Re-engineering the Corporation* spelt out what will become reality for a company of the 90s and into the next century in that companies will surely improve the way we, as American corporations, conduct business. Most interestingly, re-engineering relies heavily on information technology and automation, which is not too surprising. Indeed, many of the practices spelled out in the case studies documented in their book could not have even been thought about 15 to 20 years ago.

If one extrapolates the re-engineering fever to the field of analytical chemistry, the use of automation will concomitantly increase over the next several years as well. Such an attitude will require a fresh look at projects and situations that can be automated. The ability of laboratory personnel to 'think out of the box' will dictate their rise or fall as successful analysts.

Some of the newest challenges at Roche were discussed in this presentation.

### Laboratory automation: a critical tool in competitive, regulated industries

**Lane Gehrlein**
*Schein Pharmaceuticals, Carmel, NY, USA*

Multisource pharmaceutical companies face increasing demands for fast and efficient research and development programmes. Large domestic pharmaceutical companies are developing generic drug programmes and foreign pharmaceutical companies are also entering into the generic drug market. In order to stay competitive, companies must increase the number of new product introductions, since the market share for each product is divided up among an increasing number of competitors.

Laboratory automation has played an important role in Danbury Pharmacal's ability to develop an increasing number of new generic pharmaceutical products with minimal increases in R&D personnel. Laboratory automation has also been very effective for reducing the impact of increasing government regulatory requirements on pharmaceutical R&D programs. The laboratories employ automation in eight areas which have demonstrated a positive impact on productivity: cleaning validation-sample testing; process validation-sample testing; product assay and content uniformity; dissolution; data acquisition network; LIMS; method validation; and raw material release.

Automation in a highly regulated industry can be very productive when properly implemented and validated. Regulation can be equated with consistency which is exactly what is obtained from automation. All of the above automated procedures have been designed to deliver data which has been validated and reports which directly reference that data. No additional manipulation of data or reformatting of results is needed. Auditing of these reports is easy and fast, because the auditor knows exactly what to look for and where to find it. Automation will be a major contributor towards staying competitive in today's highly regulated industries.

### Digging out with a robot

**Milton Levenberg**
*Abbott Laboratories, Abbot Park, IL, USA*

As in every other pharmaceutical company, the chemists at Abbott Laboratories are totally dependent on a rapid return of spectroscopic data on their intermediates and synthetic products to ensure that their chemical reactions are on track. Eight years ago the chemists at Abbott often had to wait three days to a week to receive this data, and had to start new reactions without this data, hoping the previous step performed as expected. That this was an unacceptable situation was clearly recognized by the Abbott chemists and the management of the spectroscopy area.

This presentation discussed the general approach used to analyse Abbott Laboratories' needs and to provide totally automated sample preparation and running of both NMR and mass spectrometric samples. Systems by which the chemists log their own samples into our database have been developed. A Zymark robotics system does the entire sample preparation and insertion into the spectrometer. NMR spectra are plotted directly in the client's lab. The spectroscopy staff need only provide samples to the robot, fresh supplies (solvents, tubes etc.) and routine maintenance, but no actual sample handling or spectrometer operation. The effects of this automation on throughput, sample turnaround time, and cost per spectrum were presented.

### Getting smart with automation

**Richard Kramer**
*Instrumentation Development Group, Bristol-Myers Squibb Pharmaceutical Research Institute, Princeton, NJ, USA*

Laboratory productivity and quality issues have given way to business issues as the driving force behind automation and in pharmaceutical R&D in recent years. This shift brings with it unprecedented opportunity for automation where it can be related to meeting business objectives. Unfortunately, not all of the driving business issues are pretty: the budget restrictions, staffing limitations, and organizational changes that we live with today require that we thoroughly understand the role automation will play in the new climate. We have found that we are not merely charged with engineering the physical automation in the laboratories, but also engineering the strategy for the evolution of automation. We had to get smart.

In the author's R&D environment, getting smart was achieved through two distinct methods, which were discussed in depth: getting smart about automation, and getting smart from automation. The presentation considered the relationship of risk management strategies, and how education, investment and partnerships are ingredients of managing automation growth. The chemist's role as in-house integrator was examined in terms of the multitude of hats we have to wear. Also the lessons learned from implementing automation were discussed.

### Laboratory automation of the 1990s: goals, expectations and business needs

**W. Jeffrey Hurst and Robert A. Martin Jr.**
*Hershey Foods Technical Center, Hershey, PA, USA*

The laboratory of the 1990s is not immune to the trends facing American industry. For many years, laboratory professionals have felt that they could somehow be isolated from the realities of business. Laboratory automation was targeted as a panacea for a multitude of issues dealing with laboratory productivity. There has been a shift in industrial research and development, requiring laboratories to refocus their efforts and continuously evaluate the many facets of laboratory operations including financial objectives along with laboratory automation. Additionally, goals for automation technology need to be tempered with realistic expectations. This presentation focused on these issues and provided some operational solutions for organizations facing many of these same problems.

### Economic justification of automation systems in the Dow Chemical Company

**Jonathan Zieman, Paul Morabito and Ramasamy Tamilarasan**
*The Dow Chemical Company, Midland, MI, USA*

Numerous robotic automation systems have been developed and implemented within the Dow Chemical Company. These systems have found homes in production quality control laboratories, analytical laboratories, and R&D departments. The complexity of procedures automated within the company has varied, ranging from sample preparation for biological tissue analysis to polymer sample hot dissolution to polymer physical testing. Automation capabilities are currently available that can reliably perform most of the common laboratory procedures such as weighing, dilution, filtration, vial manipulations, liquid handling, incubation, and interfacing to instruments etc. The developed capabilities along with the new emerging technologies have enormous automation potential in many areas within the company.

As with all capital investments, economic justification must be evaluated for the automation impact and return on investment (ROI). It can be difficult to identify and/or quantify all the major variables needed to adequately determine the automation impact/ROI, especially for the first-time user. Lack of suitable information could result in not funding a project that would save the company money and increase productivity. ROI data acquired from several automation systems in operation over the past couple of years was used to more formally address future justifications. The issues from these systems were presented to help identify topics to consider if automation plans are in your future.

### VALIDATING LABORATORY AUTOMATION

### Evaluating the reliability of software

**Charles A. Snipes**
*FDA, Rockville, MD, USA*

Current computer industry standards for software require a written validation plan that will include the following elements:

(1) Design specifications (exactly what the program is intended to do and exactly how it is intended to do it, detailed, both high level and low level, with predetermined criteria for acceptance of the program, and descriptions of: hardware to be used, algorithms, file structure, limits and parameters to be measured, error and alarm messages, configuration, communication links among subprograms and to equipment and to other systems, and security measures).
(2) Risk/hazard analysis.

(3) Testing plans: *Developmental*, including structural analysis of all individual decision points (in the modules before integration), and testing of worst case conditions at operational limits, of alarms, of error routines, and of executable statements. *Installation*, for installation testing of the software within the environment where it has been designed to function.
(4) Evaluation of test results, particularly as to how they demonstrate that the design specifications have been met.
(5) Change control/revalidation procedures.
(6) Documentation about who is responsible for approving and for executing the validation plan.
(7) Archiving the whole of the above, including in particular all versions of the design specifications and in particular specific test results, rather than pass/fail.
(8) Especially important are the design specifications, as these are the heart of a validation.

Written development standards (for overall development) and written programming standards (for the work of individual programmers) are also of importance.

### Development and validation of an automated drug content and degradation profile analysis method for pharmaceutical tablets

**John R. Stanley**
*Pharmaceutical Technologies, SmithKline Beecham Pharmaceuticals, Hertfordshire, UK*

An automated bulk tablet drug content assay application for the HPLC analysis of pharmaceutical tablets has been developed and validated using a Zymark BenchMate Tablet Processing Workstation (TPW) with EasyFill sample collection module (EZ). The first part of the application consisted of the development and optimization of a Zymark TPW method for the homogenization of tablets and the extraction of the drug component of interest from the tablet matrix.

The second part of the application was the development of a protocol to show equivalence between the automated method and the pre-existing manual method for the extraction of drug for both bulk content drug assay and degradation profile analysis. This protocol included an assessment of TPW processed samples that were transferred to vials by the EZ for subsequent off-line HPLC analysis. The third part of the application was performance qualification validation for the equivalence protocol and interpretation of the data generated. Advantages and disadvantages of the approaches undertaken for the development and validation of the automated procedure were discussed.

### Development and validation of a Zymate PyTechnology potency robot equipped with a tablet processing workstation and on-line HPLC

**Daniel W. Barrow**
*Bristol-Myers Squibb, New Brunswick, NJ, USA*

A potency and degradant analysis robot has been successfully developed and validated for use with tablet and whole encapsulated dosage forms. The system is based on a Zymate PyTechnology robot equipped with a Tablet Processing Workstation PySection and on-line HPLC. Active drug ingredients and degradants are extracted from solid dosage forms using a high speed homogenizer. Filtration is performed by a custom filtration PySection that uses standard membrane filters and dispenses filtrate directly into the target test tube. Multiple dilutions are possible using capped test-tubes. The high capacity, 50 ml, test-tubes allow accurate dilutions and vigorous vortex mixing, virtually eliminating evaporation through the use of a robotic capping PySection. Final working solutions are automatically injected onto the on-line HPLC. Total sample capacity is increased through the use of a custom waste diversion valve which separates hazardous extraction waste from aqueous wash waste. Productivity gains of this robotic method of analysis are approximately 100% compared to manual methods of analysis.

This robotic system has been successfully used in the analysis of whole encapsulated dosage entities. The presentation described system schematics and robotic/methodology validation data including accuracy and precision.

### The validation of a Zymark Batch Dissolution System for QC and R&D analytical laboratory

**Muhammad Alburakeh, Charles DiLiberti and Mattio Citardi**
*Barr Laboratories, Pomona, NY, USA*

After considerable effort by the Batch Dissolution Project Team, the Zymark PyTechnology-Batch Dissolution Robotics Systems have been validated for use in the authors' laboratory as a fully automated dissolution testing system. They support samples provided by the QC and R&D areas, as well as process validation samples. Each system was successfully validated with USP apparatus I & II to perform on-line UV analysis, off-line HPLC vial filling and storage, single and multi sample point profiles, sample weighing and media exchange. These systems were originally installed as serial systems using telescoping shafts, filter tips and gravimetric media dispensing. The upgraded Batch Dissolution Systems are using volumetric media dispensing, standard 0·45 micron membrane filters and standard USP dissolution shafts. Some of the authors' experiences, as well as frustrations, and most importantly, successes in the transfer, pre-validation and validation of the robotics systems were presented.

### DRUG DISCOVERY

### An integrated and versatile system to rapidly determine potency, selectivity and potential for side-effects: from assay automation and data management to result

**J. Elands, C. Widmaier and M. Galvan**
*Marion Merrell Dow Research Institute, Strasbourg, France*

In a continuous effort to optimize early drug discovery the authors set out to automate the following tasks: screening new compounds against a series of receptors, uptake sites and enzymes, to establish selectivity and side-effect potential; development of HTS assays; secondary screening of HTS hits.

Task 1 is run in batch mode: a single receptor/uptake site per microtitre plate was used. Task 2 requires a temperature/$CO_2$ controlled incubator, a fluorescence and UV/Vis plate reader and a luminometer—different assay conditions are evaluated automatically and assays are rendered compatible with a fully automated system. Task 3 involves the evaluation of libraries of analogues around a hit on the original assay and a series of assays selected to assess selectivity for the target; assays of the first task are also used in this stage.

Assays could not be run during long times. So a flexible robotic system with an easy programmable GUI-based scheduler was needed. Details of the Zymark robotic system used and examples of scheduled assays were shown. Assays are continuously adapted and new assays developed for every HTS target. The end users need a flexible data management package with powerful protocol management incorporated. Compatibility with existing corporate databases is essential. ActivityBase from idBS was found to have nearly all these features included. idBS has proven to be very flexible by adding those features that were essential to our company. The architecture of ActivityBase was also discussed.

### A multi-functional microplate workstation as a strategy for changing priorities

**William S. Fillers and Diana K. Cohen**
*Sandoz Research Institute, Sandoz Pharmaceuticals Corporation, East Hanover, NJ, USA*

Shorter project lifetimes, changes in personnel and rapidly shifting priorities require a flexible approach to high capacity screening (HCS). A compact microplate robotics platform capable of operating concurrent luminescent and spectrophotometric assays was described. The operation schedule of the platform is determined by the ability to generate plates for cell based, reporter gene assays (RGA), while remaining workstation time is filled wth assays which are less constrained by supply dynamics. Sample preparation efficiency gains and conservation of the compound library are made possible by sharing sample dilution plates. Sustained output of 7500 samples per day creates the opportunity for batch or concurrent processing of primary and secondary screens. The cost advantage of rapid cycle time allows consideration of more high risk opportunities. Concurrent operation of multiple screens on a single HCS platform is an effective strategy for rapidly generating large amounts of data for decision making on competing approaches that require scarce project resources.

### Automating more for less

**M. R. Kozlowski**
*Department of Screening, Geron Corporation, Menlo Park, CA, USA*

A goal of laboratory automation is to replace human effort with robotic labour in the performance of predictable, repetitive laboratory operations. In an ideal world, this approach frees up the human element to perform more sophisticated and rewarding tasks. This goal is only realized, however, when the automation of the task takes less resources over the life of the project than simply performing the task manually. Resources include such things as human time, financial outlay (which can be converted to human time via cost per FTE), and down-time (which can be converted to financial outlay via 'burn rate'). Resource-intensive aspects of automating a task include training the robot, designing and testing (or purchasing) special robot-operated equipment, and managing the robotic system (for example, fixing 'crashes'). Automation of certain tasks is often not undertaken when the apparent resource requirement becomes too great.

This presentation discussed ways to make more tasks amenable to automation by decreasing the resource output required to perform them with a robot. Two key elements were focused upon: first, decreasing the need for specially designed equipment, and the resultant expense and delays. Many tasks can be performed by a robot using standard, or slightly modified, basic laboratory equipment. The second focus is decreasing resources lost through robot failure. Simple robot 'babysitting' techniques were also presented.

### The modular application of automation to high throughput screening

**J. V. Petersen, Jason Armstrong, Steve Hamilton and Rick Stanton**
*Amgen Incorporated, Thousand Oaks, CA, USA*

The Research Automation Technologies group at Amgen is charged with development of a coherent and responsive automation strategy to accelerate the drug discovery process. Success is measured by the timely application of the appropriate technologies to research and development applications. Due to the diversity and quick turnover in research projects, the hardware and software needs to be modular in design to facilitate the ease of development, integration and maintenance.

The cornerstone of the High Throughput Screening industry is the microtiter plate. A list of basic modular operations for an assay are barcoding, liquid handling, transporting, washing, reading and storage. Common to all of these modules are sample interaction, data interchange, external control and internal status. A variety of commercial devices and software provide a core functionality that requires further customization and enhancement to provide a fully integrated robotic system. Examples of several Amgen automated screening effors were reviewed to illustrate how this module approach can assist in the discovery of new therapeutic drugs.

### DISSOLUTION TESTING/VALIDATION

### Evolution of a rugged automated dissolution system with high sample throughput

**Richard Von Culin**
*Analytical Research & Development, Bristol-Myers Squibb Pharmaceutical Research Institute, New Brunswick, NJ, USA*

Automated dissolution testing has become a pharmaceutical industry standard with companies restructuring and trying to do more with less. Automated dissolution has been very successful for the robotics laboratory in Ananalytical R&D, New Brunswick. An on-line UV spectrophotometry robot has processed approximately 35 000 samples since 1989 and has accomplished uninterrupted dissolution runs lasting more than 50 hours. The evolution of a highly productive and rugged on-line dissolution system can be summarized in three main developmental stages: system flexibility, system relability, and efficient data management. System flexibility was obtained by programming the robot to run multiple dissolution methods. The system can run capsules, tablets, different sample potencies, and different flow cell sizes during a single dissolution run. The system has proven to be very reliable over the past six years. The only service performed routinely is preventative maintenance so the system rarely fails to complete a dissolution run. Finally, to further increase sample throughout, data management functions were automated. Custom software was written to electronically input all sample information into the System V controller and to lead the final dissolution results into the stability database. The evolution of this on-line UV dissolution robot into a rugged and productive system was presented.

### Automated dissolution testing utilizing on-line fibre optic probe UV analysis

**Leonard J. Kostek, Paul K. Aldridge, David W. Melvin, Brenda A. Williams, Karl Bratin and Sonja Sekulic**
*Analytical Research and Development, Pfizer Central Research, Groton, CT, USA*

Automated dissolution testing has become a common practice in the pharmaceutical industry. Generally, the end analysis for the dissolution test has been an ultraviolet spectrophotometric assay or an HPLC separation with ultraviolet detection. The end analyses for both methods have been performed off-line and on-line depending on the drug product. For the past 10 years, automated dissolution methodologies have encompassed vessel filling, sample dropping, medium sampling and vessel washing routines. Recent advances in fibre optic technology have complimented the traditional systems by allowing *in situ* analysis of the dissolution samples. The end analysis is performed on-line with each dissolution vessel using a robot manipulated fibre optic probe and interfaced to a conventional ultraviolet/visible spectrophotometer. No sample is removed from the dissolution vessels and no filtration is required to eliminate background interference. Various computer programs instantaneously calculate the results and transfer the data to appropriate spreadsheets.

The fibre optic method development was described, together with the testing used to validate this system. The dissolution throughput has increased considerably for samples with interfering matrix. The fibre optic end analysis has provided additional savings to the current economical automated dissolution methods. This system minimized the handling of experimental samples, providing an added measure of safety to laboratory personnel when handling very potent or sensitive materials.

### Validation of vendor-supplied systems

**Bruce Fowler, P. Michael Masterson and Clarence Kemper**
*Kemper-Masterson, Inc., Belmont, MA, USA*

The purpose of this presentation was to describe some current issues and concepts relative to vendor-supplied systems, such as LIMS, laboratory instrumentation, or robotics. The presentation incorporated practical documentation techniques for optimizing qualification and validation of vendor-supplied systems. The presentation focused on the validation premise that user firms can reduce the amount of effort and expense of qualification and validation if they rely on vendor-supplied data and information to support their systems-related activities.

The conclusions emphasized that vendor and users should work more closely together to minimize the cost and effort required to qualify and validate systems used for GLP and GMP functions.

### Establishing validation standards

**Gregg Bell**
*Quality Control—Automation and Technology, Geneva Pharmaceuticals, Inc., Broomfield, CO, USA*

In the more than 10 years that computer system validation has been an area of concern within the pharmaceutical industry, regulators, manufacturers, and system vendors have been faced with the task of determining how these systems should be documented. Although numerous articles and guidelines on validation principles have been published, the development of widely accepted validation standards has been slow to evolve.

By developing company-level validation standards, significant benefits can be realized in the areas of regulatory compliance and business performance. This paper described the benefits of establishing validation standards; described practical techniques for the development and maintenance of validation standards; and provided an example of a validation standard for a company-level computer system validation master plan document.

### ADVANCED TOPICS/DATA HANDLING/DATA MANAGEMENT

### Computer modeling of laboratory automation

**Wen-Jeng Li**
*Bristol-Myers Squibb Pharmaceutical Research Institute, Princeton, NJ, USA*

Down sizing has made a lot of scientists consider that automation will make up the loss of manpower in the laboratory. Some of them had not thought of laboratory automation previously. Some of them had some ideas about what to automate, but most of them do not know where to start, what to automate, or who to talk to.

Working as internal system integrators, we have the responsibilities to educate our scientists about laboratory automation, help them to identify potential areas, and work out solutions with them. Describing our suggestions with words and drawings alone sometimes cannot get the message across. Most scientists do not envision automation like engineers. Therefore, we started to use computer modeling software to show the scientists how ideas can work. If a picture is worth a thousand words, then moving pictures must be worth thousands times more.

With the help of life-like animations, scientists can see every step of their processes performed by an automated system. Misunderstandings can be easily corrected in this design phase. This helps to prevent financial losses and minimize wasted time due to rework.

### The integration of laboratory automation systems with LIMS in a pharmaceutical R&D environment

**Marie Di Maso, Alain LeBlanc, Carolyn Wells, Judy Wybrandts and Sam McClintock**
*Merck Frosst Centre for Therapeutic Research, Kirkland, Quebec, Canada*

To obtain regulatory approval to market a new drug product, long term stability studies are required to demonstrate chemical and physical stability during the indicated shelf life of the product. Under the current changing regulatory environment, more extensive testing and exhaustive documentation is required to support these studies. This presentation described the process of automating and integrating many of the steps required to generate the appropriate data. A LIMS system is used to set up the study protocols, log in samples for analysis, collate data, produce reports, archive the data and forecast workload. This system receives data from several sources including an automated chromatography system, balances, KF instruments, a Benchmate Tablet Processing Workstation and a MultiDose dissolution system. The process of validating and maintaining each of the systems and the associated documentation was presented. The methods development and optimization processes on the robotic systems and the authors' approach to issues encountered were discussed. Emphasis was placed on the integration of the systems and the flow of information between the system components.

### Applications of Visual Basic in laboratory automation

**Martin Echols**
*Nycomed Inc., Collegeville, PA, USA*
**and Mark F. Russo**
*Industrial BioCatalysis, Wynnewood, PA, USA*

The Visual Basic programming system for Microsoft® Windows™ is quickly becoming one of the most prominent custom application development tools for the automated laboratory. Visual Basic combines a simple programming language and an intuitive graphical user interface development environment with a wide range of built-in and third-party software libraries. Visual Basic software libraries are available for purposes such as: data acquisition, serial communications, motion control, scientific graphing, statistics, neural networks, multimedia and even laboratory robot control. This ease-of-use and continually increasing number of pertinent software libraries makes Visual Basic attractive for developing custom software components for automated laboratory systems.

The heart of any laboratory automation programming environment is its ability to communicate outside of its own boundaries. Visual Basic provides many ways to accomplish this. The authors detailed several of these communication mechanisms by discussing a variety of real laboratory automation applications using Visual Basic. These examples covered Serial and IEEE 488 communicaions, Dynamic Data Exchange (DDE), Dynamic Link Library (DLL) calls, and Object Linking and Embedding (OLE). The access of databases and local area networks, as they pertain to laboratory automation, as well as Zymark's Systems V Controller was also discussed.

### Automation of a Houillon viscometer

**Katherine S. Bonar, Robert N. Sanders and Donald R. Blevins**
*Albemarle Corporation, Baton Rouge, LA 70898, USA*

Viscosity measurement is an important part of Albemarle Corporation's R&D lubricating oil programme. Researchers can generate more samples in a given time period if sample size is small. An ISL Houillon viscometer was purchased so that small volume samples could be run. Using this manual instrument increased the workload because previously fewer large volume samples were being run on an automated viscometer. Fast turnaround is needed to allow efficient progress in research projects. It was concluded that automation would make the measurement less time-consuming and costly. Robotics would also eliminate any potential operator inattention problems due to boredom.

The Houillon was automated with Zymark PyTechnology robotics. Since this application is the first automation of a Houillon viscometer, custom designs were needed. A special pipet hand for handling small volume pipet tips was devised. A communications interface between the Zymark computer and the Houillon computer was also designed to detect instrument status and to exchange sample identification and results.

The automation has produced substantial savings in operator time. These savings will return the capital invested after running about 6000 samples. It has also resulted in the researchers receiving answers sooner, because the robot can operate overnight. Another benefit was demonstrating a robot's usefulness and potential in an R&D setting to skeptics in the organization.

### Progress toward standard commands for modular analytical instruments

**Gary W. Kramer, Peter J. Grandsard, Torsten A. Staab and Uwe Bernhoeft**
*National Institute of Standards Technology, Chemical Science and Technology Laboratory, Analytical Chemistry Division, Gaithersburg, MD, USA*

The concept of building automated analysis systems by interconnecting standard modular instruments offers great potential to reduce the costs and complexity of system integration. The Consortium on Automated Analytical Laboratory Systems (CAALS) has been working to delineate the requirements for modular instruments. Previously, CAALS has described an architecture for fully automated laboratories, developed the CAALS-I Communication Specification for instrument-to-controller messaging, and enumerated behaviours that devices should exhibit for good system citizenship. Currently, with the assistance of CAALS members and other experts in analytical and clinical chemistry automation, an attempt is being made to define a small, but powerful set of standard commands for the remote control of instruments. The approach is simple, abstract, and enabling. Since many analytical instruments are already computer-controlled and capable of storing externally defined methods, there is little reason to attempt standardization at the device command level, rather the approach is to develop a standard mechanism for invoking such stored methods. In this way, system control can be achieved without requiring instrument manufacturers to give up their existing methods of programming their instruments.

### A systematic approach to error and exception handling in automated laboratory systems using a machine vision example

**Peter J. Grandsard, Torsten A. Staab and Gary W. Kramer**
*National Institute of Standards Technology, Chemical Science and Technology Laboratory, Analytical Chemistry Division, Gaithersburg, MD, USA*

Managing errors and exceptions is a system requirement that is commonly omitted in the initial designs for laboratory automation applications. *Ad hoc* approaches for handling such unexpected events are often added during system implementation, often reducing a carefully designed control system to 'spaghetti code'. Over the past five years, the Consortium on Automated Analytical Laboratory Systems (CAALS) has developed and promoted the concept of modular instruments as building blocks for automated systems. A requirement of CAAL's modularity concept is the development of a systematic, modular approach to the handling of unplanned events.

Classically, unscheduled event handling has been carried out in three steps: event detection, event reporting, and event remediation. This process would be more modular if a fourth activity, event classification, was added between detection and remediation. In this paper, the authors presented their recent efforts using machine vision as an example to manage errors and exceptions in the operation of our Laboratory Automation System Testbed. The following issues were addressed: the classification of unplanned events; the level of synchronization between error/exception detection and remediation; and the manner by which an effected module returns to its normal operating state. Underlying the treatment of all these issues is compliance to the module behaviour requirements of the CAALS control model. Some different recovery procedures were illustrated with real examples.

### Automation integration, and regulation—*Quo Vadis?*

**Frank A. Settle**
*VMI Research Laboratories, Virginia Military Institute, Lexington, VA, USA*

What is the current state of automation in today's analytical laboratories and where is it going? Has the 'fish in–knowledge out' paradigm been implemented with automated, integrated, systems, or does automation exist only on isolated islands? This presentation outlined different approaches to laboratory automation in regulated environments, discussed the advantages and limitations of each, and cited case studies illustrating each strategy. The synergy between the flow of samples and the flow of data through automated systems was addressed.

The CAA (Contaminant Analysis Automation, Department of Energy) and CAALS (Consortium for Automated Analytical Laboratory Systems, National Institute for Standards and Technology) hardware and software standards for integration of automated devices into systems were presented. Advantages of using these standards were discussed, as well as problems encountered in their implementation.

Finally, the path to full laboratory automation via 'plug and play' standard laboratory modules (SLMS @ Scibus) was projected. The ultimate objective of this strategy is to make laboratory automation as easy as office automation through the use of standardized hardware interfaces and software drivers.

### DRUG DISCOVERY/HEALTH SCIENCE

### Automated compound preparation for high throughput screening

**Mark Beggs**
*ZENECA Pharmaceuticals, Macclesfield, UK*

High compound throughput is a key requirement in empirical screening programmes aimed at pharmaceutical lead identification. The initial compound dilution and distribution processes that form a common 'front end' to many high throughput screens can benefit significantly from effective automation. The primary need was for a system that could accommodate large numbers of microplates and perform the necessary liquid transfer operations accurately and reliably. This capability has been provided by an appropriately configured Zymark Microassay System. Deployment of this system at ZENECA Pharmaceuticals has facilitated a significant increase in compound throughput. The rationale behind automating compound dilution and distribution, the capabilities provided by the system and the resulting benefits were described.

### Design of track based automated ELISA assay for use in high throughput screening

**Jeffrey A. Guss, Joe J. Yacobucci, Janet M. Kolb and Derek J. Hook**
*Bristol-Myers Squibb, Biomolecular Research, Wallingford, CT, USA*

Drug discovery requires large quantities of samples to be assayed and evaluated in a timely fashion. The screening process contains routine mundane activities which need to be carried out in order to find novel compounds for specific therapeutic targets. Unfortunately, people are too expensive and can introduce variability to the results of an assay. So most companies interested in drug discovery automate to ensure accuracy of the results and to make better use of time. A popular assay type is the ELISA. This incorporates liquid handling, shaking, incubating, plate washing, and reading the end point. When creating an automated platform for this type of assay, an assortment of variables must be addressed if the assay is to be successfully implemented. Reagents can be light and/or temperature sensitive, and volumes of liquid to be handled, sample apacity of the system, and incubation times are just some of the variables to be considered for the science of the assay. The physical layout and overall design of the automated system has other needs. Communication, which is both active and has conformation, whether or not the system is stationary or track based, type of liquid handling station, peripherals, and space requirements are some design variables. Special requirements of the system may include: reagent cooling and/or heating, filtration, handling radioactive reagents, system waste, and external reagent pumping stations. There are two other items to consider when automating an ELISA, or any other type of assay: development time and cost. If an automation project such as this one is to be successfully designed and implemented in a timely fashion, co-operation between integrators and scientists is a necessity.

### Automated plasma radiochemical assays for quantitative functional imaging using a Zymark XP laboratory robot

**David L. Alexoff, Colleen Shea, Joanna S. Fowler, Payton King, S. John Gatley, David J. Schlyer and Alfred P. Wolf**
*Chemistry Department, Brookhaven National Laboratory, Upton, NY, USA*

Positron Emission Tomography (PET) is a nuclear medicine imaging technique that allows the quantitative observation of the time course and spatial distribution of positron-emitting radiopharmaceuticals in an organ (typically the brain or heart) of an awake human subject after intravenous injection. Combined with plasma radioactivity measurements and a validated mathematical model, this tissue radioactivity data can be used to determine specific parameters of a biochemical system (for example, neurotransmitter receptor availability) important to normal organ or tissue function. Often, multiple plasma radiochemical assays including a series of labeled metabolite correction determinations must be made for every quantitative PET procedure, requiring additional personnel working in a potentially hazardous environment.

To help overcome this potential limitation of quantitative imaging studies, a commercial laboratory robot system (Zymate PyTechnology II Laboratory Automation System) was interfaced to standard and custom laboratory equipment and programmed to perform rapid radiochemical assays necessary for plasma input function determination in quantitative PET studies in humans and baboons. A Zymark XP robot arm was used to carry out two assays: (1) the determination of total plasma radioactivity concentrations in a series of small-volume whole blood samples and (2) the determination of unchanged (parent) radiotracer in plasma using only solid phase extraction methods. Steady steady robotic throughput for determination of total plasma radioactivity in whole blood samples (0·350 ml) is 14·3 samples/hour, which includes automated centrifugation, pipetting, weighing and radioactivity counting. Robotic throughput for the assay of parent radiotracer in plasma is four to six samples/hour depending on the radiotracer. Percentage of total radioactivities present as parent radiotracers at 60 minutes post injection of $25 \pm 5{\cdot}0$ ($N = 25$), $26 \pm 6{\cdot}8$ ($N = 68$), $13 \pm 4{\cdot}4$ ($N = 30$), $32 \pm 7{\cdot}2$ ($N = 18$), $16 \pm 4{\cdot}9$ ($N = 20$), were obtained for carbon 11 labeled benztropine, raclopride, methylphenidate, SR 46349B (*trans*, 4-[(3Z)3-(2-dimethyl-amino-ethyl) oxyimino-3 (2-fluorophenyl)propen-1-yl]-phenol), and cocaine respectively in baboon plasma and $84 \pm 6{\cdot}4$ ($N = 9$), $18 \pm 11$ ($N = 10$), $74 \pm 5{\cdot}7$ ($N = 118$) and $16 \pm 3{\cdot}7$ ($N = 18$) for carbon-11 labeled benztropine, deprenyl, raclopride, and methylphenidate respectively in human plasma. The automated system has been used for more than four years for determination of unchanged tracer in plasma for seven different carbon-11 labeled compounds used routinely in the authors' laboratory. The robotic parent compound assay runs unattended and includes automated clean-up procedures that eliminates all human contact with plasma-contaminated containers.

Research for this project was supported by USDOE, OHER, and NIH Grants NS-1538, NS-15380.

### Robot-assisted synthesis of radiopharmaceuticals labeled with high activity positron emitters

**C. Brihaye, C. Lemaire and A. Luxen**
*Cylotron Research Center, University of Liege, Sart-Tilman Bat B30, B-4000 Liege, Belgium*

Positron Emission Tomography (PET) is becoming an increasingly important tool for studying physiological, biochemical and pharmacological functions at a molecular level in living man, whether in health or in disease. Radiochemical methodology constitutes the most important base for successful functioning of a PET group in the routine production and development of radiopharmaceuticals. Automatic robotic radiosynthesis is more desirable to avoid excessive radiation exposure to operatives, although the labeling of radiopharmaceuticals with high activities (>18 500 Mbq) of position emitter ($^{18}F$, $^{11}C$, $^{13}N$) by remote control is feasible. The preference for robotic radiosynthesis exists, because it appears to be more versatile, and therefore more useful for research. In the Cyclotron Research Center of the University of Liege, a robotic system has been developed base on the Zymate$^{TM}$II Laboratory Automation System (Zymark Corporation). The software control is EasyLab Plus$^{TM}$, and routines for several modules, more specific to the particular radiosynthesis were designed and fabricated in house, including a column for the separation of [$^{18}F$]fluoride from irradiated water, Sep-Pak$^{TM}$ and conventional chromatography systems, ovens for evaporation equipped with optical levels probes, a microwave oven to rapidly heat vials. Special sectors were designed for collecting fractions from a chromatographic column and automatic injection in an HPLC system, as well as for formulating the injectable solution.

Complete routine productions of [$^{18}F$] Altanserin, 4-[$^{18}F$] Fluorotropapride, 6-[$^{18}F$] Fluoro-L-Dopa and 2-[$^{18}$] Fluoro-L-Tyrosine are conducted weekly, including the labeling of the radiopharmaceuticals, the quality control and the formulation of the injectable solution.

### Automated sample preparation methods for DNA amplification by PCR

**Larry D. Sutton, Werner W. Wilke and Ronald N. Jones**
*Medical Microbiology Division, Department of Pathology, University of Iowa College of Medicine, Iowa City, IA, USA*

An automated DNA extraction system was developed which was capable of extracting DNA by several methods. Extraction was accomplished using a Zymate XP robotic system with a centrifuge, master laboratory station with remote dispenser, capping station, vortexer, centrifuge hand, 200 µl eight channel pipetting hand, one ml pipetting hand and a combination syringe-gripping hand. Peripheral equipment included Boekel minirefrigerator and freezer, and a Hitachi U-2000 double beam, scanning UV-Vis double beam spectrophotometer automated using Zymark's generic RS-232 interface. The system extracts the DNA from the sample and quality controls the preparation by measuring absorbences at 260 and 280 nm. Samples are processed in 1·5 ml screw-capped microcentrifuge tubes. Only one tube is opened at a time in order to minimize chances of cross contamination between samples. DNA amplification was accomplished in 96 well format using either a Zymate II automated PCR system with an MJ thermal controller or by reagent assembly and sample loading by a Hamilton Micro Lab 2200 and thermal cycling on a Perkin-Elmer 9600. Throughput varied according to the DNA extraction technique. The extraction efficiencies were evaluated using several different sample matrices and DNA analytes.

### A liquid handling station to rapidly assemble reagents and sample loading for DNA amplification by PCR

**Werner W. Wilke, Arzu Karabay, Ronald N. Jones and Larry D. Sutton**
*Medical Microbiology Division, Department of Pathology, University of Iowa College of Medicine, Iowa City, IA, USA*

DNA analysis based upon gene amplification by the polymerase chain reaction (PCR) continues to find new applications in medicine, agriculture, environmental analysis, and the food, pharmaceutical and biotechnology industries. Many of these applications require high throughput and reliability, especially with regard to prevention of false positives due to carry-over contamination. The performance of the Hamilton Micro Lab 2200 to assemble PCR reagents and load samples into microtubes in 96-well format for amplification on a Perkin-Elmer 9600 thermal controller was evaluated. DNA was extracted from samples using a Zymate XP system. Performance was validated by comparing to a Zymate II automated PCR system which has been evaluated over the past three years. Washing the Teflon-coated steel cannula with 5%, sodium hypochlorite followed by copious rinses with distilled water using the peristaltic pump accessory, adequately eliminated sample carry-over. The high flow rate (3 ml over 10 seconds per channel) achievable with the peristaltic pump was essential to eliminate carry-over of both target and bleach (which inhibited PCR). Up to 50% negative controls were used to assess aerosolization of target; none was detected. No build up of target DNA overtime was observed. As configured, the system is capable of assembling over 1800 PCR reactions in an eight hour shift translating into nearly 500 000 reactions per year. At this throughput, use of fixed cannula may save from $5000 to $30 000 in disposable pipette tips.

### Validation of robotic-automation of the fluorescent microsphere technique for determination of regional blood flow

**R. Hecht, S. Raab and K. Messmer**
*Institute for Surgical Research, LM-University of Munich, Germany*

Determination of regional blood flow (RBF) by means of fluorescent-labeled microspheres (FM) has been demonstrated to be an attractive alternative to conventional radioactive microspheres (RM). However, processing of tissue samples to completely extract fluorescence from the tissue is time consuming because it has to be performed manually. In addition, it presents a source of error.

The authors designed a new sample processing unit (SPU) which allows the entire processing of the tissue samples automatically. After digestion of the tissue samples by exposure to 4 N KOH at 60°C for 4 hours and vacuum-filtration, the extraction of the fluorescent dye is accomplished by adding 2(2-ethoxy-ethoxy)-ethyl-acetate and followed by centrifugation. Zymark Corporation (Hopkinton, MA, USA) provided a modified robotic system for processing the tissue sample to analyse the fluorescence of multiple colours present in each sample. For validation of the FM-method of the new automated SPU, combinations of different RM and FM (0–15 μl) were simultaneously injected into the left atrium of six pigs. For all tissue samples prepared according to an hierarchical organ dissection scheme ($N = 301$/pro animal: heart 201; brain 46; kidney 44; skeletal muscle 10), RBF was calculated based on radioactivity or fluorescence intensity, respectively.

The RBF determined by FM using the new SPU with automated measurement corresponded to RM-RBF values ($r > 0{\cdot}95$).

The automated FM method reported allows reliable determination of RBF with the advantage of automatically processing 40 samples/hour and without need of radioisotopes.

### LABORATORY WORKSTATION

### Analysis of cleaning validation samples with the Zymark BenchMate workstation

**Stephen Scypinski, Theodore Sadlowski and John Baiano**
*Hoffmann-La Roche, Inc., Nutley, NJ, USA*

The performance of cleaning validation or cleaning assessment studies is necessary to verify that a piece of pharmaceutical processing equipment is free from contamination from both active substance and cleaning agent after its use. Cleaning validation is especially important to the development arena, where it is quite common for a single blender, mill or tablet press to be utilized in the production of several products. Therefore, the cleaning of such processing equipment has become commonplace, along with the detailed protocols for the proper execution of the cleaning procedures. Because of the rigours of these procedures and the complexity of typical equipment involved, cleaning validation typically results in a multitude of samples for a single cleaning. These samples consist of either swabs obtained by wiping a prescribed area of the machine or rinse samples collected during the washing of its specified parts. While the methods used to analyse these samples are usually not very complicated, the tedium of analysing such a large amount of samples can be considerable.

The authors have found that such a procedure was an excellent candidate for automation via the Zymark BenchMate Workstation. BenchMate racks containing 16 × 100 mm test tubes with caps are given to the industrial pharmacists who perform the cleaning of the equipment following a prescribed Standard Operating Procedure (SOP). The tubes are turned to the laboratory containing swabs or rinse solutions. In the case of solutions, the racks are placed in the Zymark TurboVap for concentration followed by placement in the BenchMate for analysis. Racks containing swab samples are placed immediately in the BenchMate.

Several products have already been automated by this procedure, and the authors anticipate no further need to perform these analyses manually. The generic method and specific results for several products were presented.

### Automated analysis of oral care products by HPLC using a BenchMate sample preparation workstation

**Anthony J. Wilkes, Gisele Walraven and Jean-Marie Talbot**
*Colgate Palmolive Company, Research and Development Inc., Milmort (Herstal), B 4041 Belgium*

Automated methods have been elaborated for the analyses of several therapeutic agents of dental creams and mouthrinses. Sample weighing, dilution, mixing of liquids by cycling (mouthrinses) or vortex agitation of viscous liquids or pastes (dental creams), addition of internal standards, filtration of particulate matter, preparation of multi level calibration standards and injection onto the HPLC chromatograph was effected using the BenchMate Workstation. The BenchMate was employed in-line, thus samples and multi-level calibration standards were freshly prepared prior to injection and analysis by HPLC. Operator time was reduced to a minimum and results were furnished during unattended overnight or weekend runs. Results obtained using the BenchMate for both internal and external standard methods were in excellent agreement with those obtained using the manual procedures; RSDs were typically less than 2%.

### Automated determination of trace avermectins in waste streams

**Andrew Lange and Mark Grimaldi**
*Merck & Co., Inc., Elkton, VA, USA*

Production and environmental demands necessitated the development of a quick, sensitive assay for analysing trace avermectins in waste streams. A sensitive, automated assay that included solid-phase extraction, solvent extraction, evaporation and derivatization, followed by HPLC analysis using fluorescence detection was developed. To this end, several modifications were made to a Zymark AutoTrace™ Workstation, and custom accessories were fabricated.

To quantify these trace amounts in aqueous solution, the AutoTrace was used to extract the avermectins from waste water using solid-phase extraction. To quantify avermectins adsorbed to solids in waste waters the AutoTrace Workstation was modified to allow solvent extraction and injection of samples containing solids. A PTFE adapter was manufactured to allow stacking of a C-8 solid phase extraction column and a Florisil clean-up column in series, combining two steps in the analysis. Specialized glassware was made to accommodate 20-ml of eluate and allow the eluate to be evaporated to a residue in an HPLC vial. This new glassware required modifications to both the AutoTrace Workstation and TurboVap system. The TurboVap system was used for drying multiple samples, and a Shimadzu SIL-10A autosampler was used to automate the derivatization step.

The analysis of standards from 20 µg/l (ppb) to 1 µg/l have been tested thus far with good correlation between concentration and peak area, with a correlation coefficient ($R^2$) of 0·99. The precision ranges from 6–13% (relative standard deviation) and the detection limit is below 10 ng/l (ppt).

### Extraction of benzoylecgonine from urine samples using the Zymark RapidTrace™

**William H. Vickery, Francis X. Diamond and John de Kanel**
*National Medical Services, Willow Grove, PA, USA*

Benzoylecgonine is one of the primary metabolites excreted in the urine following cocaine exposure. It is this metabolite which is designated by SAMHSA (Substance Abuse Mental Health Service Administration formerly National Institute for Drug Abuse, NIDA) for analysis in employment and pre-employment situations to determine if cocaine has been used. Numerous methods have been published describing methods to extract benzoylecgonine from urine either by traditional liquid/liquid means or by solid phase extraction. Liquid/liquid extractions are time consuming, difficult to automate and require great analyst skill if they are to produce reproducible and accurate results. Each manufacturer of solid phase extraction cartridges has published methods to accomplish the extraction of benzoylecgonine from urine. For the purposes of evaluating the Zymark RapidTrace™ automated extraction workstation, the Applied Separations' method published for use with their Speed™ Scan ABN cartridges was chosen. The method was programmed into the RapidTrace™ software without modification. A set of 10 Zymark RapidTrace™ sample preparation units controlled by one personal computer was capable of extracting benzoylecgonine from 60 urine samples per hour. The samples were then evaporated to dryness at 40°C using a Zymark TurboVap. This step took approximately 10 minutes. The samples were then derivatized by reaction with 50 µl (BSTFA with 1% TMCS) in 50 µl ethyl acetate for 20 minutes at 70°C in a sealed tube. The samples were then transferred to autosampler vials and analysed by GC/MS. A technician would spend at least three hours preparing these extracts manually. The use of the RapidTrace frees the technician to work on more productive things such as data review for two of the three hours.

One millilitre urine samples known to be negative for cocaine metabolites were spiked with deuterated benzoylecgonine at a level of 200 ng/ml as an internal standard and with benzoylecgonine at varying levels to evaluate the recovery, linearity and carry-over of the extraction method when performed by the Zymark RapidTrace. The results from this experiment are presented in the table. The standards at 75, 150, 300 and 1000 ng/ml were used to determine back calculated levels for these standards and to quantitate the controls at 120 and 180 ng ng/ml as well as levels in various blank samples. A urine sample was extracted through the procedure after spiking it with 100 000 ng/ml benzoylecgonine. Analysis of the blank samples following this procedure was done to estimate carry-over. The procedure used was identical to the routine procedure which would be followed for real samples. No extra manipulative steps were added in an effort to minimize carry-over. The extract from the 100 000 ng/ml sample was not derivatized nor analysed by GC/MS since potential carry-over on the GC auto sampler could have confused results.

| Spike ng/ml | Calculated level, ng/ml | Ion ratio 346/240 | Ion ratio 361/240 | Ion ratio 361/346 |
|---|---|---|---|---|
| 75 | 78 | 0·1088 | 0·2844 | 2·652 |
| 150 | 142 | 0·1033 | 0·2761 | 2·673 |
| 300 | 300 | 0·0992 | 0·3224 | 3·249 |
| 1000 | 1000 | 0·0986 | 0·3088 | 3·132 |
| 120 | 129 | 0·1145 | 0·3085 | 2·694 |
| 180 | 175 | 0·1074 | 0·3253 | 3·029 |
| | 5·5 | 0·6326 | 0·9166 | 1·449 |
| 0* | 15·1 | 0·0725 | 0·3483 | 4·802 |
| | 5·2 | 0·1947 | 0·2934 | 1·507 |

* Blank extracted after the 100 000 ng/ml spiked sample extraction.

Under SAMHSA guidelines an affirmative cut-off of 150 ng/ml is used to administratively differentiate positive samples from negative ones. Irrespective of other data, samples containing benzoylecgonine below this administrative level are reported to clients as negative. In addition to this quantitative test, three ion ratios must be within 20% of values determined for a standard. Direct quantitation of the blank following the 100 000 ng/ml spike yields 15 ng/ml or 0·015% carry-over. Some of this level is derived from the background noise. The blanks extracted after relatively low levels of benzoylecgonine quantitate to approximately 5 ng/ml, for example. These levels are all far below the administrative cut off of 150 ng/ml. In addition to this, however, the ion ratio test fails for all the blank runs. Benzoylecgonine cannot even be identified in these samples, therefore.

### AUTOMATION AND COMBINATORIAL CHEMISTRY

### Second-generation robotic synthesizer for peptide, pseudopeptide and non-peptide libraries

**Jean A. Boutin and Jean-Luc Fauchere**
*Division des Peptides, Institut de Recherches Servier, 92150 Suresnes, France*

A second-generation peptide library synthesizer has been constructed around a Zymark robotic arm. In order to provide new solutions for the ever-growing field of combinatorial libraries, it has been designed to handle gram quantities of resin for the solid phase synthesis of potentially any type of library.

To ensure a wide molecular diversity, a series of 24 building blocks was used and therefore, the robot synthesis station is a table hooked with 25 (5 columns of 5) reactors, slaved to individual gas and waste valves. The 25th reactor serves as a control permitting the independent but concomitant synthesis of a single defined product which is closely analysed by HPLC, mass spectrometry and nuclear magnetic resonance. Since libraries (i.e. defined mixtures of oligomers made up of at least 24 building blocks) are extremely complex mixtures, with up to several millions of individual chemicals, a minimum of 1 g (i.e. $2 \times 10^7$ beads) per reactor vessel was considered the absolute requirement to ensure the proper and complete synthesis of random tetra- to penta peptide libraries, or of higher oligomer libraries with fixed positions.

Designed to perform the so-called mix-and-divide strategy, the robot comprises a 1 l mixing chamber that functions as a mixer between each step of the combinatorial synthesis. This chamber is filled and emptied by a CombiTip-based hand that ensures the proper and total transfer of the bead suspension. Finally the instrument comprises also a section for automatically dissolving the starting materials (not limited to natural or exotic amino acids), thus ensuring the maximum stability of the chemicals before coupling. This section comprises five sets of 25 tubes in which the dry building blocks are stocked and five additional devices for large quantities of common reagents. The robot comprises a set of valves and hands that handle up to 10 different liquids used in the chemical reactions or as washing solvents.

This instrument is a unique tool for the iterative synthesis of defined libraries. More than a 100 different peptide, pseudopeptide and non-peptide libraries (over 20 million compounds altogether) have been synthesized in the authors' laboratory in the past two years leading to ligands of therapeutically relevant targets active in the micro to nanomolar ranges.

### Automated preparation and purification of amides

**R. Michael Lawrence, Olga M. Fryszman, Michael A. Poss, Scott. A. Biller and Harold N. Weller**
*Bristol-Myers Squibb Company, Princeton, NJ, USA*

With ever-increasing pressures on the pharmaceutical industry to bring innovative products to the marketplace more quickly, Bristol-Myers Squibb Company is exploring automated organic synthesis as a means to accelerate the drug discovery process. To this end, we have developed an automated solution phase procedure to rapidly prepare amides. Utilizing a Zymark BenchMate Workstation, up to 100 reactions can be set up and the resulting products purified by exploiting the solid phase extraction capabilities of the BenchMate Workstation. The procedures for reaction set-up, product purification, analytical sample preparation and electronic data handling were described in this presentation.

### Automated combinatorial chemistry on solid phase

**John F. Cargill**
*Ontogen Corporation, Carlsbad, CA, USA*

High speed solid phase synthesis of diverse, non-peptidyl small organic molecules in a spatially dispersed combinatorial library (SDCL) is accomplished with proprietary automation systems called OntoBLOCK and OntoCODE. These systems support reagent introduction and removal, agitation, inert atmosphere, temperature control, pressure control and vacuum drying. In the OntoBLOCK system, reaction blocks are processed by a series of custom-built, task specific workstations organized in an assembly line fashion. Compounds are cleaved from each of the 96 individual reaction vessels of the reaction block system directly into the wells of a standard 96-well microtitre plate. These plates, with one compound per well, are readily accepted onto Ontogen's automated high throughput screening (HTS) systems. Client/server database software for inventory control, experiment design, instrument control, HTS data analysis and structure activity relationship (SAR) studies is written in Visual Basic and Oracle7. Synthesis of compounds in the OntoBLOCK system is driven by instrument control language (ICL) scripts which are generated by the experiment planning software and integrated within the database system. In the OntoCODE system, miniature reaction capsules are electronically coded with a solid state radio frequency tag and undergo a number of split and recombine synthetic steps. The OntoCODE tag is electronically read after each step to maintain a reaction histogram. After the synthetic steps are completed, the discrete compounds are cleaved from the coded capsules into standard 96-well microtitre plates for HTS, with one compound per well. In both systems, an automated mass spectrometer processes the microtitre plates and produces spectra for each compound.

### A totally integrated approach to combinatorial chemistry

**Peter L. Myers**
*CombiChem, Inc., La Jolla, CA, USA*

Combinatorial chemistry is emerging as one of a series of technology advances, which over the next decade, could potentially provide a more efficient and successful approach to drug discovery. As with the advent of many new technologies there has been very significant evolution from the initial creative, but complex, methodologies which validated the concept to a growing requirement for a simplifying strategy to leverage its full potential. The real value of the technology to the medicinal chemist, who is ultimately responsible for selecting and optimizing lead structures, lies in the ability to maximize the information gained in screening combinatorial libraries of molecules against a wide range of biological targets, and to patent file composition claims to molecules of most interest, in a traditional manner.

As these are essential requirements, it is obvious that the focus in library design and synthesis should be on multiparallel synthesis of single compounds (discretes) in multimilligram quantities with appropriate QC methods for purity and confirmation of identity. This is most readily achieved by making smaller libraries and simultaneously eliminates the need for complicated tagging and/or deconvolution strategies which are not always successful and may complicate the chemical processes.

However, proportionately larger numbers of such libraries will now be necessary, to ensure that we exploit the potential diversity open to us. They must be well designed both for synthesis and more specifically *content*. Ideally the synthetic methodology should also be capable of simple automation to minimize manpower resource.

Thus CombiChem's intention as a combinatorial chemistry company is to develop an integrated package comprising state of the art combinatorial library synthesis incorporating computational software design and instrumentation which together will form the major infrastructure towards optimal library design and synthesis.

### DIVERSOMER$^{TM}$ technology and recent advances in automated synthesis

**S. Hobbs DeWitt, N. Halim, E. Hogan, A. W. Czarnik**
*Parke-Davis Pharmaceutical Research, Division of Warner-Lambert Company, Ann Arbor, MI, USA*
**A. MacDonald and R. Ramage**
*University of Edinburgh, Department of Chemistry, Edinburgh, UK*

Advances in molecular biology and automation have revolutionized the testing of compounds for drug discovery. Biologists at Parke-Davis can now test up to 500 000 samples in one month. Therefore, traditional methods for chemically synthesizing compounds one-by-one can no longer meet the demands of mass screening. The ability to synthesize multiple compounds in a simultaneous fashion is the goal of Parke-Davis' DIVERSOMER$^{TM}$ technology. Automation of routine and labour-intensive steps of chemistry is critical to advancing combinatorial and high throughput synthesis. Although automation has been extensively used in clinical chemistry and high throughput screening, it has not been integrated into synthetic chemistry laboratories. This is largely due to the fact that chemistry requires individual and precise manipulations. The objectives of automation in chemistry are not only speed and productivity but also precision in sample transfers and manipulations.

DIVERSOMER$^{TM}$ technology enables the high throughput synthesis of organic compounds using both manual and automated methods. The system design incorporates a modular approach employing robotic automation to achieve parallel processing of 40 intermediates or products. Automation and integration has been achieved by modifying and exploiting commercial software and hardware products. Representative hardware and software includes the DIVERSOMER$^{TM}$ apparatus, a Tecan robotic sample processor, Microsoft Excel, and MDL software. The combination of these tools enables a full range of operations necessary for the generation and testing of compound libraries for combinatorial chemistry and molecular diversity programs.

